# Association Between HDL Cholesterol Changes and Cardiovascular Event Risk: A Nationwide Health Screening Cohort in Japan

**DOI:** 10.3390/healthcare14131959

**Published:** 2026-07-02

**Authors:** Sunhwa Kim, Sunyeup Kim, Nang kyeong Lee, Seung won Lee

**Affiliations:** 1 Department of MetaBioHealth, School of Medicine, Sungkyunkwan University, Suwon 16419, Republic of Korea; kshcj132@gmail.com; 2 Department of Precision Medicine, School of Medicine, Sungkyunkwan University, Suwon 16419, Republic of Korea; paul5506@g.skku.edu (S.K.); skdrud3717@skku.edu (N.k.L.); 3 Department of Artificial Intelligence, Sungkyunkwan University, Suwon 16419, Republic of Korea; 4 Personalized Cancer Immunotherapy Research Center, School of Medicine, Sungkyunkwan University, Suwon 16419, Republic of Korea; 5 Department of Family Medicine, Kangbuk Samsung Hospital, School of Medicine, Sungkyunkwan University, Seoul 03181, Republic of Korea

**Keywords:** HDL cholesterol, HDL-C trajectory, cardiovascular disease, cohort study, longitudinal change, risk stratification, myocardial infarction, heart failure, atrial fibrillation, epidemiology

## Abstract

**Background:** Low high-density lipoprotein cholesterol (HDL-C) is an established marker of cardiovascular risk. However, HDL-C levels may change over time in relation to metabolic status, lifestyle factors, and medication use, and the cardiovascular implications of longitudinal HDL-C changes remain incompletely understood. **Methods:** We conducted a retrospective cohort study using the JMDC Claims Database, including 3,387,924 adults who underwent at least two health checkups between 2005 and 2021. Participants were categorized into four groups based on HDL-C changes between two time points: persistently low, low to normal, normal to low, and persistently normal. The primary outcome was incident composite cardiovascular disease (CVD), including myocardial infarction, angina pectoris, stroke, heart failure, and atrial fibrillation. Cox proportional hazards models were used to estimate hazard ratios (HRs) with adjustment for demographic and clinical covariates. **Results:** During a mean follow-up of 4.3 years, persistently low HDL-C was associated with the highest risk of composite CVD compared with persistently normal HDL-C (HR 1.15, 95% CI 1.12–1.19). Both Low-to-Normal and Normal-to-Low groups also showed elevated risks (HR 1.10, 95% CI 1.06–1.14; HR 1.14, 95% CI 1.10–1.19, respectively). The strongest association was observed for myocardial infarction, whereas the association with stroke was modest and less consistent after full adjustment. **Conclusions:** Longitudinal changes in HDL-C were associated with cardiovascular risk after adjustment for cardiometabolic factors and medication use. Persistently low HDL-C conferred the greatest risk, and serial HDL-C patterns may provide additional information for cardiovascular risk assessment beyond a single baseline measurement.

## 1. Introduction

Cardiovascular disease (CVD) remains the leading cause of morbidity and mortality worldwide, imposing a substantial global public health burden [1]. Among the well-established cardiovascular risk factors, dyslipidemia occupies a central role, with decades of research underscoring the importance of lipid management in primary and secondary prevention strategies [2]. While LDL-C has long been recognized as the primary lipid treatment target [3], growing evidence suggests that the residual cardiovascular risk persisting even under optimal LDL-C lowering cannot be fully explained by LDL-C alone [4].

High-density lipoprotein cholesterol (HDL-C) has traditionally been regarded as a major protective factor against atherosclerotic cardiovascular disease (ASCVD). Large-scale epidemiological studies, including the landmark Framingham Heart Study, demonstrated a robust inverse association between HDL-C levels and the incidence of coronary heart disease (CHD), independent of other established risk factors including LDL-C [5,6]. Subsequent meta-analyses corroborated these findings, firmly establishing low HDL-C as an independent predictor of adverse cardiovascular outcomes [7]. The protective mechanisms underlying HDL-C’s cardiovascular benefit are multifaceted. HDL particles facilitate reverse cholesterol transport (RCT), removing excess cholesterol from peripheral tissues and macrophage foam cells within the arterial wall, thereby mitigating the atherosclerotic burden [8]. Beyond RCT, HDL exerts pleiotropic anti-inflammatory, anti-oxidative, anti-thrombotic, and anti-apoptotic effects on the vascular endothelium and myocardium [9,10]. Specifically, HDL inhibits endothelial cell adhesion molecule expression, suppresses monocyte chemotaxis, and promotes nitric oxide-mediated vasodilation, collectively dampening vascular inflammation [11].

Despite this body of evidence, the HDL hypothesis has been challenged in recent years. Multiple large randomized controlled trials employing pharmacological interventions that substantially raise HDL-C, including cholesteryl ester transfer protein (CETP) inhibitors and extended-release niacin, failed to reduce major adverse cardiovascular events [12]. Furthermore, Mendelian randomization studies and genetic analyses have questioned whether HDL-C is causally protective or merely a marker of underlying metabolic health [13]. Additionally, paradoxical observations have emerged suggesting that extremely high HDL-C levels may be associated with increased rather than decreased cardiovascular and all-cause mortality, presenting a U-shaped or J-shaped relationship in some large cohort studies [14]. These apparent contradictions have prompted a paradigm shift from focusing on HDL-C quantity to examining HDL functionality, including cholesterol efflux capacity, as a more meaningful determinant of cardiovascular risk [15].

Despite extensive research into baseline HDL-C levels, comparatively little attention has been directed toward the impact of longitudinal changes in HDL-C on cardiovascular outcomes. HDL-C is not a static biomarker; it fluctuates over time in response to changes in lifestyle, body weight, metabolic status, pharmacological therapies, and systemic inflammation. A prior study examining changes in HDL-C from baseline demonstrated that patients who experienced improvements in HDL-C during follow-up had more favorable cardiovascular outcomes, suggesting that the direction and magnitude of HDL-C trajectory, not simply its baseline value, carries independent prognostic significance. Expanding on this concept, a large population-based cohort study using serial HDL-C measurements demonstrated that persistently low HDL-C and a change from normal to low HDL-C were independently associated with increased risks of multiple gastrointestinal cancers, with effect modification by sex, smoking status, and age [16]. These findings underscore the importance of evaluating HDL-C as a dynamic, longitudinally changing biomarker rather than a single baseline measurement. Whether analogous longitudinal changes in HDL-C confer differential risks for cardiovascular diseases, particularly coronary artery disease (CAD) encompassing composite ASCVD, heart failure (HF), and atrial fibrillation (AF), has not been systematically evaluated in a large population-based setting.

CAD, encompassing myocardial infarction, angina pectoris, and ischemic stroke, is the dominant manifestation of ASCVD and represents the principal cause of cardiovascular death globally [1]. In parallel, HF and AF are rapidly growing public health crises in aging societies [17]. While ASCVD risk has traditionally been linked to elevated atherogenic lipoproteins and reduced HDL-C, the lipid-related determinants of HF and AF are considerably less understood. Regarding HF, HDL-C has been proposed to exert direct myocardial protection through anti-inflammatory and anti-apoptotic pathways, in addition to reducing coronary atherosclerosis that can ultimately precipitate ischemic cardiomyopathy [18]. For AF, a paradoxical inverse association between cholesterol levels and AF risk has been consistently observed, wherein higher cholesterol levels including HDL-C appear to confer protection against AF development, possibly through membrane-stabilizing effects on cardiomyocyte ion channels [19,20]. In this context, previous large-scale population-based studies have suggested that lipid fractions may show heterogeneous associations across cardiovascular outcomes, with atherogenic lipid markers generally showing positive associations with ASCVD and HF, whereas lipid associations with AF appear more complex or paradoxical [21]. These findings support the need to assess individual cardiovascular outcomes separately when evaluating lipid biomarkers, rather than relying solely on composite endpoints.

Low HDL-C frequently coexists with other lipid and metabolic abnormalities, including elevated triglycerides, insulin resistance, and other features of atherogenic dyslipidemia. These interrelated abnormalities may contribute to cardiovascular risk and highlight the importance of accounting for coexisting lipid-related and cardiometabolic risk factors when evaluating HDL-C trajectories [22,23].

To date, evidence remains limited regarding whether longitudinal changes in HDL-C levels are differentially associated with incident cardiovascular outcomes, including myocardial infarction, angina pectoris, stroke, HF, and AF, after adjustment for available lipid-related and cardiometabolic risk factors. Therefore, the present study aimed to evaluate the association between longitudinal HDL-C changes and incident composite CVD outcomes and their individual components, with a particular focus on whether maintaining or recovering normal HDL-C levels modifies long-term cardiovascular risk in a primary prevention setting.

## 2. Materials and Methods

### 2.1. Ethics Statement and Data Source

This study used the JMDC Claims Database, one of the largest claims databases in Japan [24]. The database integrates medical and pharmacy claims (including diagnoses, procedures, and prescriptions) with annual health check-up records from multiple employer-based health insurance societies. The initial study population comprised approximately 5 million individuals who underwent at least two health checkups between January 2005 and July 2022. The JMDC population primarily consists of company employees and their dependents, which may limit the generalizability of the findings to elderly or unemployed populations.

### 2.2. Study Population

From the JMDC Claims Database, we identified individuals who had undergone at least two recorded annual health checkups between January 2005 and July 2022. The index date was defined as the date of the second health checkup. For inclusion in the analysis, the second health checkup, defined as the index health checkup, was required to occur between January 2008 and July 2021 to ensure that at least three years of claims history before the index date were available for identifying prior cardiovascular disease. At this point, the change in HDL-C from the first health checkup was determined, and follow-up was initiated.

Participants were classified into four groups based on the longitudinal change in HDL-C level between the first and second health checkups: persistently low HDL-C (Low-to-Low), low to normal HDL-C (Low-to-Normal), normal to low HDL-C (Normal-to-Low), and persistently normal HDL-C (Normal-to-Normal). Low HDL-C was defined as HDL-C less than 40 mg/dL for all participants, in accordance with the Japan Atherosclerosis Society criteria. Normal HDL-C was defined as HDL-C of 40 mg/dL or greater.

We excluded individuals with a prior diagnosis of cardiovascular disease, including ASCVD, HF, or AF, within the three years before the index date. The interval between the first and second health checkups was restricted to within three years. Among individuals with at least two health screenings (n = 4,436,834), we further excluded those with missing HDL-C values at either the first or second health checkup (n = 341,019), missing BMI, LDL-C, systolic blood pressure, diastolic blood pressure, triglyceride, or fasting blood glucose (n = 351,267), missing age or sex (n = 0), age < 18 years (n = 0), and follow-up duration of less than one year (n = 356,624).

After applying these exclusion criteria and restricting the analysis to participants with available covariate information required for the fully adjusted models, the final analytic cohort comprised 3,387,924 individuals. These participants were classified as Low-to-Low (n = 84,680), Low-to-Normal (n = 64,276), Normal-to-Low (n = 62,739), and Normal-to-Normal (n = 3,176,229), with the Normal-to-Normal group serving as the reference.

### 2.3. Exposures and Outcomes

The primary exposure was the longitudinal change in HDL-C level between the first and second annual health checkups, categorized into the four groups described above. The primary outcome was the first occurrence of composite cardiovascular disease (CVD), defined as myocardial infarction, angina pectoris, stroke, HF, or AF. Outcomes were identified using disease-specific ICD-10 diagnosis codes combined with relevant procedure or treatment codes, where applicable, to improve outcome specificity in the claims database. Detailed outcome definitions are provided in Supplementary Table S1.

**Figure 1:**
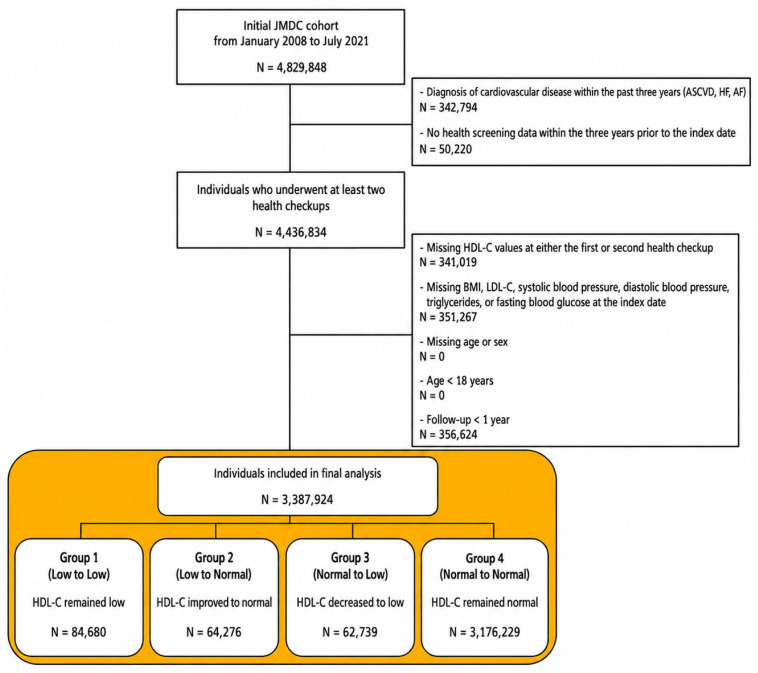
Participant selection flowchart.

The event date was defined as the date of the first occurrence of any outcome after the index date. Each outcome component was also analyzed individually as a secondary outcome. Participants were followed from the index date until the first occurrence of any composite CVD event, death, or the end of the study period (July 2022), whichever came first (Figure 1).

### 2.4. Covariates

Baseline covariates were collected at the index date from health checkup records and claims data. Health checkup variables included age, sex, body mass index (BMI), physical activity, smoking status, alcohol consumption frequency, LDL-C, triglycerides, systolic blood pressure, diastolic blood pressure, and fasting glucose. Age was analyzed as both a continuous variable and a categorical variable (<40, 40–64, and ≥65 years). BMI was categorized, with obesity defined as a BMI ≥ 25 kg/m^2^. Smoking status was classified as current smoker or non-smoker, and alcohol consumption frequency was categorized as every day, sometimes, or rarely. Physical activity was categorized as sufficient or insufficient.

Comorbid conditions were additionally assessed using claims data, including hypertension, diabetes mellitus, chronic kidney disease, hyperthyroidism, hypothyroidism, chronic liver disease, and autoimmune diseases. Comorbidities were defined as at least two recorded diagnoses at separate clinical visits, based on ICD-10 codes. Medication use was assessed using prescription claims and included antihypertensive, antidiabetic, and lipid-lowering medications. We calculated the first-to-second health checkup interval in years and included it as a continuous covariate.

### 2.5. Statistical Analysis

Baseline characteristics are presented as mean ± standard deviation for continuous variables and number with percentage for categorical variables. Follow-up time was defined as the time from the index health checkup to the first occurrence of each outcome, death, or the end of follow-up (July 2022), whichever came first. The Cox models used follow-up time from the index health checkup as the analysis time scale. Incidence rates were calculated per 1000 person-years with 95% confidence intervals (CIs).

We evaluated the association between HDL-C change groups and each cardiovascular outcome using Cox proportional hazards models, with the persistently normal HDL-C group (Normal-to-Normal) as the reference. Three models were constructed: Model 1 was unadjusted; Model 2 was adjusted for age and sex; and Model 3 was fully adjusted for age, sex, BMI, physical activity, smoking status, alcohol consumption, LDL-C, triglycerides, systolic blood pressure, diastolic blood pressure, fasting glucose, comorbidities, medication use, and the interval between the first and second health checkups. Missing values for lifestyle variables were handled as separate missing categories in the primary analysis. Hazard ratios (HRs) and 95% CIs were estimated for each model. The proportional hazards assumption was assessed using log-minus-log survival plots and the results are shown in Supplementary Figure S1. Fine–Gray subdistribution hazard models were also fitted for each cardiovascular outcome to account for the competing risk of death before cardiovascular event occurrence.

Subgroup analyses were conducted by sex and age group (<40, 40–64, and ≥65 years) to examine potential effect modification. In addition, a pre-specified subgroup analysis was performed among individuals with a history of lipid-lowering medication use.

As sensitivity analyses, we performed three additional analyses. First, we repeated the primary Cox proportional hazards analyses after including participants with follow-up shorter than one year. Second, among participants with available serum creatinine data, we additionally adjusted for eGFR to assess the influence of kidney function. Third, we conducted a complete-case analysis excluding participants with missing values for lifestyle covariates, including physical activity, smoking status, and alcohol consumption.

All statistical analyses were performed using SAS version 9.4 (SAS Institute Inc., Cary, NC, USA), and a two-sided *p* < 0.05 was considered statistically significant.

## 3. Results

### 3.1. Baseline Characteristics of Study Participants

A total of 3,387,924 individuals were included in the final analysis and categorized into four groups according to HDL-C change status. The majority of participants were aged 40–64 years across all groups, accounting for approximately 65–70% of the study population, while individuals aged ≥65 years represented a small proportion (approximately 2–3%). The proportion of younger individuals aged <40 years was slightly higher in the Normal-to-Low group compared to the other groups.

Male participants were predominant in the Low-to-Low, Low-to-Normal, and Normal-to-Low groups, whereas the sex distribution was more balanced in the Normal-to-Normal group. The distribution of lifestyle factors showed modest differences between groups. The proportion of individuals with sufficient physical activity was relatively low overall but slightly higher in the Normal-to-Normal group. Similarly, current smoking and frequent alcohol consumption were less common in the Normal-to-Normal group compared to the other groups.

Notable differences were observed in cardiometabolic profiles. The prevalence of obesity was highest in the Low-to-Low group and lowest in the Normal-to-Normal group. Triglyceride levels were markedly higher in the Low-to-Low and Normal-to-Low groups than in the Normal-to-Normal group. Blood pressure and fasting glucose levels were also higher in the HDL-C abnormality groups than in the Normal-to-Normal group.

Regarding comorbid conditions, the prevalence of hypertension, diabetes, and other systemic diseases was consistently higher in the Low-to-Low group and lowest in the Normal-to-Normal group. Medication use also differed across groups. Antihypertensive, antidiabetic, and lipid-lowering medication use were more frequent in the HDL-C abnormality groups than in the Normal-to-Normal group. The checkup interval was similar across HDL-C trajectory groups and was restricted to within three years by design. Follow-up duration was similar across all groups, with a mean of approximately 4.3 years. Standardized mean differences for baseline characteristics are provided in Supplementary Table S2. Annual counts of cardiovascular events during the study period are provided in Supplementary Table S3 (Table 1).

### 3.2. Cumulative Incidence of Cardiovascular Outcomes

**Figure 2:**
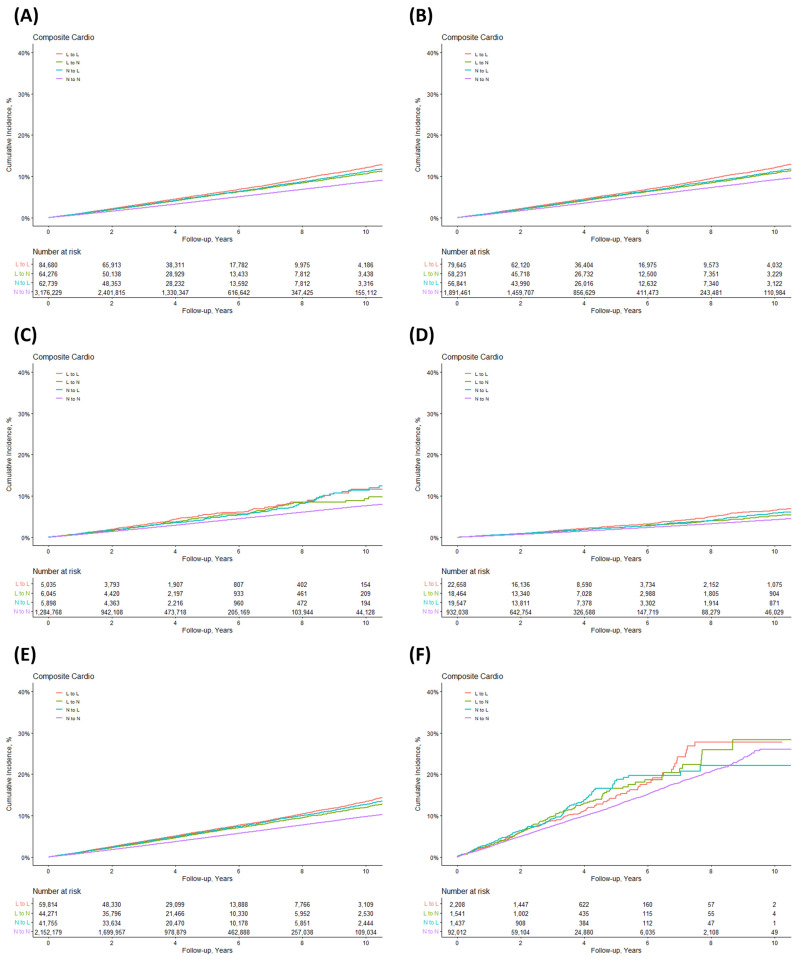
Cumulative incidence of cardiovascular outcomes according to HDL-C trajectory groups. The cumulative incidence of cardiovascular outcomes across HDL-C trajectory groups is shown for (**A**) all participants, and stratified by sex: (**B**) male and (**C**) female, and by age groups: (**D**) <40 years, (**E**) 40–64 years, and (**F**) ≥65 years. The number of individuals at risk is listed below each panel. *p*-values were derived from log-rank tests comparing the four trajectory groups.

To examine the temporal pattern of cardiovascular event accumulation across HDL-C trajectory groups, Kaplan–Meier curves for event-free survival were constructed for each outcome of interest (Figure 2A). Clear separation in event-free survival probabilities was observed across all four trajectory groups for composite CVD, myocardial infarction, angina pectoris, stroke, and HF throughout the follow-up period (all log-rank *p* < 0.001). The Low-to-Low group consistently exhibited the lowest event-free survival, followed by the Normal-to-Low and Low-to-Normal groups, whereas the Normal-to-Normal group maintained the highest event-free survival at all time points.

Notably, divergence between groups was evident from the earliest follow-up interval and widened progressively over time, suggesting that the adverse cardiovascular impact of persistently low HDL-C manifests early and accumulates over time.

With respect to individual outcomes, the separation between the Low-to-Low group and the Normal-to-Normal reference group was most pronounced for myocardial infarction and angina pectoris, reflecting the particularly strong association between sustained HDL-C deficiency and coronary atherosclerotic burden. For stroke and HF, group separation was similarly evident from early follow-up, although the magnitude of divergence was comparatively attenuated relative to coronary outcomes. In contrast, AF demonstrated a less pronounced separation across trajectory groups compared with coronary outcomes and HF. This pattern was consistent with the weak association observed in the subsequent regression analyses.

Sex-stratified analyses revealed broadly consistent patterns of group separation in both male and female participants (Figure 2B,C). However, among female participants, the survival curves for atrial fibrillation demonstrated more pronounced separation between the Low-to-Low and Normal-to-Normal groups compared to male participants, providing early visual evidence of the sex-specific association identified in the regression analyses.

Age-stratified analyses demonstrated robust group separation across all three age categories (Figure 2D–F). Among participants younger than 40 years, the Low-to-Low group showed the steepest early decline in event-free survival, suggesting that the cardiovascular burden of persistently low HDL-C may be particularly pronounced in younger individuals. In the oldest age group (≥65 years), while overall event rates were higher across all groups, reflecting the increased baseline cardiovascular risk in this population, the relative separation between trajectory groups was comparatively attenuated, consistent with the pattern observed in subsequent regression analyses.

Survival curves for participants with a history of lipid-lowering medication use are presented in Supplementary Figure S2. Additional Kaplan–Meier analyses stratified by sex and age are presented in Supplementary Figures S3–S7. While the overall pattern of separation across HDL-C trajectory groups was preserved in this subgroup, the magnitude of divergence between groups appeared attenuated compared to the main analysis, suggesting potential heterogeneity in underlying risk profiles within this higher-risk subgroup.

### 3.3. HDL-C Trajectory and Risk of Incident Cardiovascular Disease

The results of Cox proportional hazards regression analyses are presented in Table 2. Across all models, the Low-to-Low group showed the highest risk of composite CVD compared with the Normal-to-Normal reference group. In the fully adjusted model, the association was attenuated but remained statistically significant (HR 1.15, 95% CI 1.12–1.19). The Low-to-Normal and Normal-to-Low groups also had higher risks of composite CVD than the reference group, with adjusted HRs of 1.10 (95% CI 1.06–1.14) and 1.14 (95% CI 1.10–1.19), respectively. These findings indicate that the excess risk associated with unfavorable HDL-C trajectories persisted after adjustment for demographic factors, cardiometabolic parameters, comorbidities, and medication use, although the magnitude of association was modest after full adjustment. The log-minus-log survival plot for composite CVD showed generally parallel curves across HDL-C trajectory groups, suggesting no substantial violation of the proportional hazards assumption for the primary outcome (Figure S1).

For individual cardiovascular outcomes, the association was most pronounced for myocardial infarction. The Low-to-Low group had more than a two-fold higher risk of myocardial infarction after full adjustment (HR 2.22, 95% CI 1.68–2.93). The Normal-to-Low group also showed a significantly increased risk of myocardial infarction (HR 1.63, 95% CI 1.11–2.40), whereas the Low-to-Normal group showed a borderline association (HR 1.46, 95% CI 0.99–2.14). Angina pectoris showed a similar pattern, with all three non-reference groups demonstrating significantly elevated risks after full adjustment: Low-to-Low (HR 1.78, 95% CI 1.39–2.28), Low-to-Normal (HR 1.58, 95% CI 1.17–2.14), and Normal-to-Low (HR 1.72, 95% CI 1.27–2.33). HF was also consistently associated with unfavorable HDL-C trajectories, with adjusted HRs of 1.43 (95% CI 1.35–1.52), 1.23 (95% CI 1.14–1.33), and 1.34 (95% CI 1.24–1.44) for the Low-to-Low, Low-to-Normal, and Normal-to-Low groups, respectively.

In contrast, associations with stroke were weaker after full adjustment. The Low-to-Low group was not significantly associated with stroke (HR 1.03, 95% CI 0.99–1.08), whereas the Low-to-Normal and Normal-to-Low groups showed small but statistically significant increases in risk (HR 1.06, 95% CI 1.01–1.11 and HR 1.08, 95% CI 1.02–1.13, respectively). AF showed a comparatively weak association with HDL-C trajectory; after full adjustment, only the Low-to-Low group remained statistically significant (HR 1.09, 95% CI 1.01–1.17), whereas the other groups were not significantly associated. In the Fine–Gray competing risk models accounting for death as a competing event, the overall pattern of associations was similar to that observed in the fully adjusted Cox models (Table 3).

In sex-stratified analyses, findings were broadly consistent across male and female participants for most outcomes (Supplementary Tables S4 and S5). In sex-stratified analyses for AF, elevated risk was observed among female participants in the Low-to-Low group (HR 1.62, 95% CI 1.10–2.37), whereas the Low-to-Normal group was not significantly associated with AF risk (HR 1.13, 95% CI 0.73–1.74). The Normal-to-Low group showed a borderline association among female participants (HR 1.48, 95% CI 1.00–2.20). These associations were not clearly observed among male participants.

In age-stratified analyses for composite CVD, associations varied across age groups (Supplementary Tables S6–S8). Among participants younger than 40 years, the Low-to-Low group was associated with an increased risk of composite CVD (HR 1.18, 95% CI 1.07–1.29), whereas the Low-to-Normal (HR 1.05, 95% CI 0.94–1.17) and Normal-to-Low (HR 1.11, 95% CI 0.99–1.23) groups were not statistically significant. Among participants aged 40–64 years, all three non-reference groups showed significantly elevated risks of composite CVD: Low-to-Low (HR 1.16, 95% CI 1.12–1.20), Low-to-Normal (HR 1.10, 95% CI 1.06–1.14), and Normal-to-Low (HR 1.15, 95% CI 1.10–1.20). In contrast, no statistically significant association was observed among participants aged 65 years or older.

Subgroup analyses among participants with lipid-lowering medication use were broadly consistent with the primary findings for composite CVD, although the associations differed across individual outcomes (Supplementary Tables S9–S14). In this subgroup, all three non-reference groups showed elevated risks of composite CVD: Low-to-Low (HR 1.17, 95% CI 1.08–1.26), Low-to-Normal (HR 1.12, 95% CI 1.03–1.22), and Normal-to-Low (HR 1.21, 95% CI 1.10–1.32). However, AF was not significantly associated with HDL-C trajectory in this subgroup: Low-to-Low (HR 1.06, 95% CI 0.89–1.26), Low-to-Normal (HR 1.11, 95% CI 0.92–1.35), and Normal-to-Low (HR 1.05, 95% CI 0.85–1.30).

Three separate sensitivity analyses were conducted by independently applying the following conditions: including participants with a follow-up period shorter than one year, restricting the analysis to participants with available serum creatinine data and additionally adjusting for eGFR, and excluding participants with missing information on smoking or drinking status. Across these sensitivity analyses, the associations between HDL-C trajectory and composite CVD were generally consistent with the main analysis. When participants with a follow-up period shorter than one year were included, the HRs were 1.16 (95% CI, 1.12–1.20) for the Low-to-Low group, 1.10 (95% CI, 1.06–1.14) for the Low-to-Normal group, and 1.14 (95% CI, 1.10–1.19) for the Normal-to-Low group. When the analysis was restricted to participants with available serum creatinine data and additionally adjusted for eGFR, the corresponding HRs were 1.14 (95% CI, 1.09–1.19), 1.12 (95% CI, 1.06–1.18), and 1.17 (95% CI, 1.11–1.23), respectively. After excluding participants with missing information on smoking or drinking status, the corresponding HRs were 1.14 (95% CI, 1.11–1.18), 1.10 (95% CI, 1.06–1.14), and 1.13 (95% CI, 1.09–1.18), respectively. Results for individual cardiovascular outcomes, including heart failure, atrial fibrillation, angina pectoris, stroke, and myocardial infarction, are presented in the Supplementary Tables (Supplementary Tables S15–S17).

## 4. Discussion

In this large population-based cohort of Japanese adults, longitudinal changes in HDL-C were associated with the risk of several cardiovascular outcomes after adjustment for demographic factors, cardiometabolic parameters, comorbidities, medication use, and the interval between health checkups. Participants with persistently low HDL-C generally showed the highest risk of composite CVD, myocardial infarction, angina pectoris, and HF, although the magnitude of association was attenuated after full adjustment. The associations were most pronounced for coronary outcomes and HF, whereas the association with stroke was modest and less consistent. The association with AF was weak overall and varied across subgroup analyses.

A key finding is that HDL-C is more appropriately interpreted as a longitudinal marker of cardiometabolic risk rather than as a single static measurement. Conventional risk assessment based on one-time HDL-C values does not adequately capture persistent or newly developed HDL-C abnormalities. Previous studies have reported similar patterns [25,26]. Persistent low HDL-C and declines over time have been linked to increased disease risk, while improvements in HDL-C were associated with better outcomes [27]. Our findings extend this evidence in a much larger population and across multiple cardiovascular endpoints. Biologically, HDL plays a central role in reverse cholesterol transport and exerts anti-inflammatory, anti-oxidative, and endothelial protective effects [8,28]. However, HDL-C concentration does not necessarily reflect HDL functionality. Impaired cholesterol efflux capacity, altered anti-inflammatory or anti-oxidative properties, and dysfunctional HDL particles may contribute to residual cardiovascular risk even when HDL-C concentration appears quantitatively normal. Because the JMDC health checkup data included HDL-C concentration but not functional HDL assays, we could not determine the underlying mechanism. Sustained or recurrent low HDL-C may therefore contribute to cumulative vascular damage. The lower risk observed in the Low-to-Normal group compared with the Low-to-Low group suggests that recovery is beneficial, although not sufficient to fully normalize risk.

Persistently low HDL-C was strongly associated with atherosclerotic cardiovascular disease. The risks of myocardial infarction and angina pectoris were highest in the Low-to-Low group, consistent with prior evidence linking low HDL-C to atherosclerotic cardiovascular risk [5,7]. The Framingham Heart Study and subsequent large-scale analyses have established low HDL-C as an independent predictor of cardiovascular events. Our findings add that longitudinal HDL-C patterns may provide additional information beyond a single baseline value. In contrast, associations with stroke were modest after full adjustment, and the Low-to-Low group was not significantly associated with stroke risk. This suggests that the relationship between HDL-C trajectory and cardiovascular outcomes may differ across vascular phenotypes, with stronger associations for coronary outcomes than for stroke.

All non-reference groups showed increased risk of HF. HDL may influence HF through mechanisms beyond atherosclerosis, including effects on myocardial metabolism, inflammation, and oxidative stress [29]. The higher prevalence of metabolic risk factors in the low HDL-C groups supports this interpretation. These conditions are closely linked to both reduced HDL-C and HF risk. The similar risk levels in the Low-to-Normal and Normal-to-Low groups indicate that low HDL-C observed at either measurement may be associated with subsequent HF risk.

The association between HDL-C trajectory and AF was weaker and less consistent than that observed for coronary outcomes and HF. In the overall analysis, only the Low-to-Low group showed a small increase in AF risk after full adjustment, whereas the Low-to-Normal and Normal-to-Low groups were not significantly associated with AF. Subgroup analyses suggested possible heterogeneity by sex, but these findings were not consistent across trajectory groups and should be interpreted cautiously. This pattern is compatible with previous studies reporting paradoxical or heterogeneous associations between lipid levels and incident AF [27,30]. Higher cholesterol levels have sometimes been associated with lower AF risk. Proposed mechanisms include membrane stabilization and differences in inflammatory pathways. Additionally, the relatively young and healthy JMDC population may have limited the number of AF events. Moreover, asymptomatic or paroxysmal AF may be underdiagnosed in administrative claims data, which could have attenuated the observed association between HDL-C trajectory and incident AF. Therefore, the absence of association should be interpreted cautiously rather than as definitive evidence of no relationship.

Low HDL-C is often accompanied by other metabolic abnormalities, including elevated triglycerides and insulin resistance [31]. This clustering may partly explain the observed associations. However, several associations remained significant after adjustment for multiple covariates, including LDL-C, triglycerides, blood pressure, fasting glucose, comorbidities, and medication use [32]. This suggests that HDL-C trajectories provide additional prognostic information beyond traditional risk factors.

These findings have several clinical implications. First, repeated HDL-C measurements may help identify individuals with unfavorable cardiometabolic risk profiles and higher subsequent cardiovascular risk. However, this should be interpreted within the context of current lipid management guidelines. The Japan Atherosclerosis Society Guidelines define low HDL-C as a component of dyslipidemia and recognize its association with ASCVD risk, while also indicating that HDL-C alone is not a target for drug intervention [33]. Similarly, contemporary ACC/AHA and ESC/EAS guidelines primarily emphasize absolute cardiovascular risk assessment and LDL-C-centered lipid management, including statin-based LDL-C reduction according to baseline risk [34]. Although lipid-lowering therapies may influence HDL-C levels to varying degrees, the established cardiovascular benefit of statins and other lipid-lowering agents is primarily mediated through reductions in LDL-C and other atherogenic lipoproteins rather than through HDL-C raising itself. Accordingly, in the present study, baseline lipid-lowering medication use was adjusted for in the multivariable models and additionally examined in subgroup analyses, while changes in medication use during follow-up could not be fully captured.

Therefore, longitudinal HDL-C changes should be interpreted as markers of cardiovascular risk patterns rather than as direct pharmacological targets. Second, normalization of HDL-C does not fully eliminate risk. Patients with previous low HDL-C may still require continued comprehensive risk management. Third, the differential associations across individual outcomes highlight the need to evaluate cardiovascular outcomes separately when studying lipid markers.

This study has several strengths. The large sample size allowed robust evaluation across multiple cardiovascular outcomes. The use of repeated HDL-C measurements enabled assessment of longitudinal changes. Adjustment for a wide range of covariates reduced potential confounding. However, several limitations should be considered. The JMDC database mainly includes working-age individuals, limiting generalizability. Although outcome definitions combined diagnosis codes with hospitalization records and procedure or imaging information where applicable, outcome misclassification cannot be fully excluded because external validation data were unavailable. Residual confounding cannot be excluded, particularly for lifestyle factors and medication use. Socioeconomic variables beyond employment-based insurance status were not available in the JMDC database and thus could not be included in the analyses. Although remnant cholesterol and non-HDL cholesterol may be relevant to residual cardiovascular risk, these lipid parameters could not be evaluated because total cholesterol was not available in the database. Time-varying changes in covariates, including medication use and cardiometabolic profiles, were not accounted for during follow-up. Missing values in lifestyle variables were handled using missing categories, which may have introduced residual bias. HDL-C was measured at only two time points, which limits the ability to capture more complex longitudinal patterns, such as stable, transient, gradually declining, recovering, or fluctuating HDL-C trajectories. As a result, the four trajectory groups used in this study should be interpreted as simplified two-point change categories rather than reflecting comprehensive long-term HDL-C trajectories. Although the first-to-second health checkup interval was restricted to within three years and adjusted for in the multivariable models, cumulative exposure to low HDL-C could not be fully quantified. Formal interaction tests were not performed for all subgroup analyses, and multiple outcomes and subgroup analyses were evaluated. Therefore, subgroup-specific findings, particularly those for AF, should be considered exploratory. Reverse causality due to subclinical cardiovascular disease, systemic inflammation, or other preclinical illnesses may also have contributed to declining HDL-C levels before clinically recognized cardiovascular events. Finally, this observational design does not allow causal inference. HDL-C may reflect underlying metabolic status rather than directly mediating risk.

## 5. Conclusions

In this large population-based cohort, longitudinal changes in HDL-C were associated with incident cardiovascular outcomes after adjustment for cardiometabolic factors and medication use. Individuals with persistently low HDL-C had the highest risk of composite CVD, myocardial infarction, and HF, whereas low-to-normal and normal-to-low HDL-C groups also remained at modestly elevated risk compared with individuals with persistently normal HDL-C. The associations differed across individual outcomes, with stronger associations for myocardial infarction and HF and weaker or less consistent associations for stroke and AF. These findings suggest that serial HDL-C patterns may provide additional information for cardiovascular risk assessment beyond a single baseline measurement. However, given the observational nature of this study, these results should be interpreted as evidence of risk stratification rather than causality.

## Figures and Tables

**Table 1:** Baseline demographic characteristics of each population group.

Variable	Group 1 Low-to-Low (n = 84,680)	Group 2 Low-to-Normal (n = 64,276)	Group 3 Normal-to-Low (n = 62,739)	Group 4 Normal-to-Normal (n = 3,176,229)
**Age (Mean ± SD)**	45.13 ± 10.02	44.60 ± 10.25	44.01 ± 10.46	44.68 ± 10.87
**Age category**				
<40	22,658 (26.76)	18,464 (28.73)	19,547 (31.16)	932,038 (29.34)
40–64	59,814 (70.64)	44,271 (68.88)	41,755 (66.55)	2,152,179 (67.76)
≥65	2208 (2.61)	1541 (2.40)	1437 (2.29)	92,012 (2.90)
**Sex (%)**				
Male	79,645 (94.05)	58,231 (90.60)	56,841 (90.60)	1,891,461 (59.55)
Female	5035 (5.95)	6045 (9.40)	5898 (9.40)	1,284,768 (40.45)
**Sufficient physical activity (%)** ^1^				
Yes	12,579 (14.85)	10,744 (16.72)	9515 (15.17)	594,212 (18.71)
No	62,045 (73.27)	45,829 (71.30)	45,043 (71.79)	2,210,674 (69.60)
Missing	10,056 (11.88)	7703 (11.98)	8181 (13.04)	371,343 (11.69)
**Obesity (%)** ^2^				
BMI ≥ 25	52,945 (62.52)	32,268 (50.20)	33,945 (54.11)	734,256 (23.12)
**Smoking (%)**				
Smoker	41,426 (48.92)	27,258 (42.41)	25,898 (41.28)	765,354 (24.10)
Non-smoker	39,852 (47.06)	34,285 (53.34)	33,809 (53.89)	2,288,645 (72.06)
Missing	3402 (4.02)	2733 (4.25)	3032 (4.83)	122,230 (3.85)
**Alcohol consumption (%)**				
Every Day	10,002 (11.81)	10,584 (16.47)	9807 (15.63)	697,316 (21.95)
Sometimes	29,106 (34.37)	22,252 (34.62)	22,090 (35.21)	1,046,123 (32.94)
Rarely	36,519 (43.13)	24,498 (38.11)	23,584 (37.59)	1,101,423 (34.68)
Missing	9053 (10.69)	6942 (10.80)	7258 (11.57)	331,367 (10.43)
**Systemic diseases (%)** ^3^				
Hypertension	4855 (5.73)	3610 (5.62)	3424 (5.46)	110,163 (3.47)
Diabetes	8364 (9.88)	6378 (9.92)	5090 (8.11)	176,264 (5.55)
Chronic kidney disease	427 (0.50)	258 (0.40)	282 (0.45)	8,769 (0.28)
Hyperthyroidism	409 (0.48)	454 (0.71)	285 (0.45)	22,949 (0.72)
Hypothyroidism	540 (0.64)	440 (0.68)	409 (0.65)	25,911 (0.82)
Chronic liver disease	1263 (1.49)	1038 (1.61)	849 (1.35)	29,071 (0.92)
Autoimmune diseases	2009 (2.37)	1767 (2.75)	1505 (2.40)	84,469 (2.66)
**Medication use (%)** ^4^				
Antihypertensive medication	12,896 (15.23)	8989 (13.99)	8488 (13.53)	317,233 (9.99)
Antidiabetic medication	6199 (7.32)	4062 (6.32)	3308 (5.27)	78,022 (2.46)
Lipid-lowering medication	9714 (11.47)	7415 (11.54)	6128 (9.77)	211,991 (6.67)
**Cardiometabolic parameters (Mean ± SD)** ^5^				
LDL-Cholesterol, mg/dL	122.97 ± 32.37	130.90 ± 31.95	121.47 ± 31.94	120.45 ± 31.26
Triglyceride, mg/dL	243.93 ± 190.40	161.15 ± 104.97	233.97 ± 203.97	98.52 ± 70.11
Systolic Blood Pressure, mmHg	124.35 ± 15.40	123.37 ± 15.41	123.09 ± 15.25	118.85 ± 16.08
Diastolic Blood Pressure, mmHg	77.87 ± 11.60	77.12 ± 11.64	76.69 ± 11.59	73.48 ± 11.83
Fasting Blood Glucose, mg/dL	102.91 ± 29.30	99.95 ± 24.39	100.00 ± 26.01	93.99 ± 16.69
**Checkup interval, years (Mean ± SD)** ^6^	1.02 ± 0.27	1.03 ± 0.28	1.03 ± 0.28	1.04 ± 0.30
**Follow up, years (Mean ± SD)**	4.30 ± 2.70	4.35 ± 2.79	4.35 ± 2.76	4.31 ± 2.76

^1.^ Physical activity, smoking status, and alcohol consumption were obtained from routine health checkup records. ^2.^ BMI ≥ 25.0 kg/m^2^ was defined as obesity. ^3.^ Systemic diseases were defined based on repeated diagnoses recorded at separate clinical visits. ^4.^ Medication use was assessed using prescription claims at baseline. ^5.^ Cardiometabolic parameters were obtained from health checkup records at the index date. ^6.^ Checkup interval indicates the time between the first and second health checkups within three years.

**Table 2:** Incidence rates and hazard ratios for cardiovascular outcomes across HDL-C trajectory groups.

				Model 1		Model 2		Model 3	
	Person	Events	Incidence Rate per 1000 Person Years (95% CI)	HR (95%CI)	*p*-Value	HR (95%CI)	*p*-Value	HR (95%CI)	*p*-Value
**Composite CVD**									
Group 1	84,680	4415	11.95 (11.61–12.31)	1.40 (1.36–1.44)	<0.001	1.32 (1.28–1.36)	<0.001	1.15 (1.12–1.19)	<0.001
Group 2	64,276	2978	10.63 (10.26–11.02)	1.24 (1.20–1.29)	<0.001	1.20 (1.16–1.25)	<0.001	1.10 (1.06–1.14)	<0.001
Group 3	62,739	3028	11.03 (10.65–11.43)	1.29 (1.24–1.34)	<0.001	1.27 (1.23–1.32)	<0.001	1.14 (1.10–1.19)	<0.001
Group 4	3,176,229	113,725	8.53 (8.48–8.58)	REF		REF		REF	
**Myocardial Infarction**									
Group 1	84,680	69	0.19 (0.15–0.24)	5.83 (4.52–7.52)	<0.001	4.19 (3.25–5.41)	<0.001	2.22 (1.68–2.93)	<0.001
Group 2	64,276	30	0.11 (0.08–0.15)	3.34 (2.30–4.83)	<0.001	2.53 (1.75–3.67)	<0.001	1.46 (0.99–2.14)	0.053
Group 3	62,739	30	0.11 (0.08–0.16)	3.40 (2.35–4.92)	<0.001	2.65 (1.83–3.84)	<0.001	1.63 (1.11–2.40)	0.012
Group 4	3,176,229	426	0.03 (0.03–0.04)	REF		REF		REF	
**Angina Pectoris**									
Group 1	84,680	82	0.22 (0.18–0.28)	4.32 (3.44–5.44)	<0.001	3.11 (2.48–3.92)	<0.001	1.78 (1.39–2.28)	<0.001
Group 2	64,276	49	0.17 (0.13–0.23)	3.41 (2.55–4.56)	<0.001	2.61 (1.95–3.48)	<0.001	1.58 (1.17–2.14)	0.003
Group 3	62,739	48	0.17 (0.13–0.23)	3.40 (2.54–4.56)	<0.001	2.67 (1.99–3.58)	<0.001	1.72 (1.27–2.33)	0.001
Group 4	3,176,229	681	0.05 (0.05–0.06)	REF		REF		REF	
**Stroke**									
Group 1	84,680	2259	6.12 (5.87–6.37)	1.10 (1.05–1.15)	<0.001	1.13 (1.08–1.18)	<0.001	1.03 (0.99–1.08)	0.185
Group 2	64,276	1689	6.03 (5.75–6.33)	1.08 (1.03–1.14)	0.001	1.13 (1.07–1.18)	<0.001	1.06 (1.01–1.11)	0.025
Group 3	62,739	1679	6.12 (5.83–6.42)	1.10 (1.05–1.15)	<0.001	1.16 (1.11–1.22)	<0.001	1.08 (1.02–1.13)	0.004
Group 4	3,176,229	74,055	5.56 (5.52–5.60)	REF		REF		REF	
**Heart Failure**									
Group 1	84,680	1257	3.40 (3.22–3.60)	2.28 (2.15–2.41)	<0.001	1.96 (1.85–2.07)	<0.001	1.43 (1.35–1.52)	<0.001
Group 2	64,276	721	2.57 (2.39–2.77)	1.72 (1.60–1.85)	<0.001	1.53 (1.42–1.65)	<0.001	1.23 (1.14–1.33)	<0.001
Group 3	62,739	764	2.78 (2.59–2.99)	1.86 (1.73–2.00)	<0.001	1.69 (1.57–1.82)	<0.001	1.34 (1.24–1.44)	<0.001
Group 4	3,176,229	19,806	1.49 (1.47–1.51)	REF		REF		REF	
**Atrial fibrillation**									
Group 1	84,680	748	2.03 (1.89–2.18)	1.44 (1.34–1.55)	<0.001	1.16 (1.08–1.25)	<0.001	1.09 (1.01–1.17)	0.035
Group 2	64,276	489	1.75 (1.60–1.91)	1.24 (1.13–1.36)	<0.001	1.05 (0.96–1.15)	0.313	1.01 (0.93–1.11)	0.766
Group 3	62,739	507	1.85 (1.69–2.01)	1.31 (1.20–1.43)	<0.001	1.14 (1.04–1.24)	0.004	1.07 (0.98–1.17)	0.148
Group 4	3,176,229	18,757	1.41 (1.39–1.43)	REF		REF		REF	

Hazard ratios (HRs) with 95% confidence intervals (CIs) were estimated using Cox proportional hazards models, with the Normal-to-Normal group (Group 4) as the reference category. Group 1, Low-to-Low; Group 2, Low-to-Normal; Group 3, Normal-to-Low; Group 4, Normal-to-Normal. Incidence rates are presented per 1000 person-years with 95% confidence intervals. Model 1 is univariable; Model 2 is adjusted for age and sex; Model 3 is the fully adjusted multivariable model, adjusted for age, sex, body mass index, physical activity, smoking status, alcohol consumption, LDL-cholesterol, triglycerides, systolic blood pressure, diastolic blood pressure, fasting glucose, comorbidities, medication use, and checkup interval. Medication use included antihypertensive, antidiabetic, and lipid-lowering medications.

**Table 3:** Fine–gray competing risk analysis for cardiovascular outcomes across HDL-C trajectory groups.

				Competing Risk Model	
	Person	Events	Incidence Rate per 1000 Person Years (95% CI)	sHR (95%CI)	*p*-Value
**Composite CVD**					
Group 1	84,680	4415	11.95 (11.61–12.31)	1.15 (1.12–1.19)	<0.001
Group 2	64,276	2978	10.63 (10.26–11.02)	1.10 (1.06–1.14)	<0.001
Group 3	62,739	3028	11.03 (10.65–11.43)	1.14 (1.10–1.19)	<0.001
Group 4	3,176,229	113,725	8.53 (8.48–8.58)	REF	
**Myocardial Infarction**					
Group 1	84,680	69	0.19 (0.15–0.24)	2.22 (1.66–2.96)	<0.001
Group 2	64,276	30	0.11 (0.08–0.15)	1.46 (0.97–2.18)	0.067
Group 3	62,739	30	0.11 (0.08–0.16)	1.63 (1.12–2.37)	0.011
Group 4	3,176,229	426	0.03 (0.03–0.04)	REF	
**Angina Pectoris**					
Group 1	84,680	82	0.22 (0.18–0.28)	1.78 (1.39–2.28)	<0.001
Group 2	64,276	49	0.17 (0.13–0.23)	1.58 (1.15–2.19)	0.005
Group 3	62,739	48	0.17 (0.13–0.23)	1.72 (1.25–2.35)	0.001
Group 4	3,176,229	681	0.05 (0.05–0.06)	REF	
**Stroke**					
Group 1	84,680	2259	6.12 (5.87–6.37)	1.03 (0.98–1.08)	0.202
Group 2	64,276	1689	6.03 (5.75–6.33)	1.06 (1.01–1.11)	0.027
Group 3	62,739	1679	6.12 (5.83–6.42)	1.08 (1.02–1.13)	0.005
Group 4	3,176,229	74,055	5.56 (5.52–5.60)	REF	
**Heart Failure**					
Group 1	84,680	1257	3.40 (3.22–3.60)	1.43 (1.35–1.52)	<0.001
Group 2	64,276	721	2.57 (2.39–2.77)	1.23 (1.14–1.33)	<0.001
Group 3	62,739	764	2.78 (2.59–2.99)	1.34 (1.24–1.44)	<0.001
Group 4	3,176,229	19,806	1.49 (1.47–1.51)	REF	
**Atrial fibrillation**					
Group 1	84,680	748	2.03 (1.89–2.18)	1.08 (1.00–1.17)	0.040
Group 2	64,276	489	1.75 (1.60–1.91)	1.01 (0.92–1.11)	0.782
Group 3	62,739	507	1.85 (1.69–2.01)	1.07 (0.98–1.17)	0.153
Group 4	3,176,229	18,757	1.41 (1.39–1.43)	REF	

Subdistribution hazard ratios (sHRs) with 95% confidence intervals (CIs) were estimated using Fine–Gray competing risk models, treating death before cardiovascular event occurrence as a competing event. Incidence rates are presented per 1000 person-years with 95% CIs. The Fine–Gray models were adjusted for the same covariates as Model 3 in the Cox proportional hazards analyses.

## Data Availability

The data used in this study are not publicly available due to privacy and legal restrictions. The Japan Medical Data Center (JMDC) data are available under license from JMDC and are accessible upon reasonable request and with permission from JMDC.

## Supplementary Materials

The following supporting information can be downloaded at: https://www.mdpi.com/article/10.3390/healthcare14131959/s1.

## Abbreviations

The following abbreviations are used in this manuscript:

HDL-C	High-density lipoprotein cholesterol
LDL-C	Low-density lipoprotein cholesterol
CVD	Cardiovascular disease
ASCVD	Atherosclerotic cardiovascular disease
CAD	Coronary artery disease
HF	Heart failure
AF	Atrial fibrillation
HR	Hazard ratio
CI	Confidence interval
BMI	Body mass index
RCT	Reverse cholesterol transport
CETP	Cholesteryl ester transfer protein
VLDL	Very low-density lipoprotein
IDL	Intermediate-density lipoprotein
JMDC	Japan Medical Data Center
ICD-10	International Classification of Diseases, 10th Revision
