# Supplementary material for: Association Between HDL Cholesterol Changes and Cardiovascular Event Risk: A Nationwide Health Screening Cohort in Japan
Source: Healthcare (Basel). 2026 Jul 2;14(13):1959. doi: 10.3390/healthcare14131959 (PMC13361876; doi:10.3390/healthcare14131959)

healthcare

MDPI

Supplementary Materials

Association Between HDL Cholesterol Changes and Cardiovascular Event Risk: a nationwide Health Screening cohort in Japan

Sun hwa Kim[1†], Sun yeup Kim[2†], Nang kyeong Lee[2], Seung won Lee[1,2,3,4,5*]

Table S1. Detailed outcome definitions, including ICD-10 diagnosis codes and procedure/treatment codes

Outcome	Diagnosis codes	Additional definition criteria
Composite cardiovascular disease	I21, I22; I20; I60, I61, I62, I63; I50, I11.0; I48	Defined as the first occurrence of myocardial infarction, angina pectoris, stroke, heart failure, or atrial fibrillation.
Myocardial infarction	I21, I22	Hospital admission with a myocardial infarction diagnosis and percutaneous coronary intervention or coronary artery bypass grafting within 1 week.
Angina pectoris	I20	Hospital admission with an angina pectoris diagnosis and percutaneous coronary intervention or coronary artery bypass grafting within 1 week.
Stroke	I60, I61, I62, I63	Stroke diagnosis with magnetic resonance imaging or computed tomography within 1 month.
Heart failure	I50, I11.0	Hospital admission with a heart failure diagnosis.
Atrial fibrillation	I48	At least one inpatient claim or at least two outpatient claims with an atrial fibrillation diagnosis.

ICD-10, International Classification of Diseases, 10th Revision.

 https://doi.org/10.3390/healthcare14131959

Table S2. Standardized mean differences for baseline characteristics according to HDL-C trajectory groups

Characteristic	Low-to-Low vs Normal-to-Normal	Low-to-Normal vs Normal-to-Normal	Normal-to-Low vs Normal-to-Normal
Age, years	0.043	0.008	0.063
Age category, <40 years	0.058	0.014	0.039
Age category, 40–64 years	0.062	0.024	0.026
Age category, ≥65 years	0.018	0.031	0.038
Sex, male	0.896	0.769	0.769
Physical activity, sufficient	0.103	0.052	0.095
Physical activity, insufficient	0.081	0.037	0.048
Physical activity, missing	0.006	0.009	0.041
Body mass index ≥25 kg/m²	0.868	0.586	0.671
Smoking, current smoker	0.534	0.396	0.373
Smoking, non-smoker	0.527	0.394	0.383
Smoking, missing	0.009	0.020	0.048
Alcohol consumption, every day	0.273	0.140	0.162
Alcohol consumption, sometimes	0.030	0.036	0.048
Alcohol consumption, rarely	0.174	0.071	0.061
Alcohol consumption, missing	0.008	0.012	0.036
Hypertension	0.108	0.103	0.096
Diabetes	0.163	0.164	0.102
Chronic kidney disease	0.037	0.022	0.029
Hyperthyroidism	0.031	0.002	0.035
Hypothyroidism	0.021	0.015	0.019
Chronic liver disease	0.053	0.063	0.041
Autoimmune diseases	0.018	0.006	0.017
Antihypertensive medication	0.158	0.123	0.11
Antidiabetic medication	0.227	0.189	0.146
Lipid-lowering medication	0.168	0.170	0.113
Low-density lipoprotein cholesterol, mg/dL	0.079	0.331	0.032
Triglycerides, mg/dL	1.014	0.702	0.888
Systolic blood pressure, mmHg	0.349	0.287	0.271
Diastolic blood pressure, mmHg	0.375	0.310	0.274
Fasting blood glucose, mg/dL	0.374	0.285	0.275
Checkup interval, years	0.07	0.034	0.034
Follow-up, years	0.004	0.014	0.014

Values are absolute standardized mean differences calculated using the Normal-to-Normal group as the reference. For categorical variables, each category was treated as a binary indicator. An absolute standardized mean difference ≥0.10 was considered to indicate a meaningful imbalance.

Table S3. Annual number of incident composite cardiovascular disease events during follow-up

Group	Total events	0.5y	1y	2 y	3 y	4 y	5 y	6 y	7 y	8 y	9 y	10 y	≥10 y
**Composite CVD**													
Group1	4,415	417	431	900	736	564	403	272	194	189	120	75	114
Group2	2,978	324	267	590	486	405	283	192	133	110	74	50	64
Group3	3,028	316	322	572	472	397	295	180	145	119	83	59	68
Group4	113,725	12,131	11,829	22,954	18,734	14,742	10,237	7,138	4,984	4,044	2,731	1,867	2,334
**Myocardial Infarction**													
Group1	69	5	8	16	11	7	10	1	2	2	1	3	3
Group2	30	5	4	4	3	7	2	1	1	0	1	0	2
Group3	30	5	7	4	1	2	2	2	1	4	1	0	1
Group4	426	41	43	77	70	62	30	27	23	16	13	15	9
**Angina Pectoris**													
Group1	82	9	10	15	18	6	4	7	6	2	1	1	3
Group2	49	7	4	8	6	8	2	5	3	2	2	0	2
Group3	48	3	4	8	6	8	4	7	2	2	0	2	2
Group4	681	68	58	139	104	88	67	47	34	26	23	18	9
**Stroke**													
Group1	2,259	209	226	462	372	304	217	136	94	90	64	36	49
Group2	1,689	182	160	343	272	229	156	113	73	65	31	31	34
Group3	1,679	161	190	309	289	220	167	88	86	64	43	32	30
Group4	74,055	7,948	8,076	15,126	12,196	9,563	6,624	4,642	3,143	2,543	1,683	1,131	1,380
**Heart Failure**													
Group1	1,257	95	107	259	212	159	113	83	70	64	35	20	40
Group2	721	65	58	130	126	105	75	47	31	26	27	14	17
Group3	764	82	67	150	116	93	76	50	35	22	28	22	23
Group4	19,806	1,620	1,819	3,847	3,315	2,748	1,969	1,268	934	821	556	388	521
**Atrial fibrillation**													
Group1	748	99	80	148	123	88	59	45	22	31	19	15	19
Group2	489	65	41	105	79	56	48	26	25	17	13	5	9
Group3	507	65	54	101	60	74	46	33	21	27	11	3	12
Group4	18,757	2,454	1,833	3,765	3,049	2,281	1,547	1,154	850	638	456	315	415

Values are presented as the number of incident events during follow-up according to HDL-C trajectory group. Composite cardiovascular disease was defined as the first occurrence of myocardial infarction, angina pectoris, stroke, heart failure, or atrial fibrillation.

Table S4. Association between HDL-C trajectory groups and cardiovascular disease risk, stratified by sex: male participants

				Model1		Model2		Model3		Model4	
	**Person**	**Events**	**Incidence Rate per 1,000 Person Years (95% CI)**	**HR (95%CI)**	**P-value**	**HR (95%CI)**	**P-value**	**HR (95%CI)**	**P-value**	**sHR (95%CI)**	**P-value**
**Composite CVD**											
Group1	79,645	4,197	12.01 (11.65–12.38)	1.31 (1.27–1.35)	<0.001	1.32 (1.28–1.36)	<0.001	1.15 (1.12–1.19)	<0.001	1.15 (1.11–1.19)	<0.001
Group2	58,231	2,756	10.73 (10.34–11.14)	1.17 (1.13–1.22)	<0.001	1.20 (1.16–1.25)	<0.001	1.10 (1.06–1.14)	<0.001	1.10 (1.05–1.14)	<0.001
Group3	56,841	2,799	11.14 (10.74–11.56)	1.22 (1.17–1.26)	<0.001	1.27 (1.23–1.32)	<0.001	1.15 (1.10–1.19)	<0.001	1.15 (1.10–1.19)	<0.001
Group4	1,891,461	76,213	9.16 (9.10–9.23)	REF		REF		REF		REF	
**Myocardial Infarction**											
Group1	79,645	67	0.19 (0.15–0.24)	4.00 (3.09–5.18)	<0.001	4.07 (3.15–5.28)	<0.001	2.18 (1.64–2.89)	<0.001	2.17 (1.62–2.91)	<0.001
Group2	58,231	29	0.11 (0.08–0.16)	2.35 (1.61–3.42)	<0.001	2.46 (1.68–3.58)	<0.001	1.43 (0.97–2.11)	0.070	1.43 (0.95–2.15)	0.085
Group3	56,841	30	0.12 (0.08–0.17)	2.48 (1.71–3.59)	<0.001	2.66 (1.83–3.85)	<0.001	1.65 (1.12–2.43)	0.010	1.65 (1.13–2.40)	0.009
Group4	1,891,461	401	0.05 (0.04–0.05)	REF		REF		REF		REF	
**Angina Pectoris**											
Group1	79,645	82	0.23 (0.19–0.29)	3.04 (2.41–3.82)	<0.001	3.13 (2.49–3.94)	<0.001	1.81 (1.41–2.32)	<0.001	1.81 (1.41–2.32)	<0.001
Group2	58,231	48	0.19 (0.14–0.25)	2.42 (1.80–3.24)	<0.001	2.57 (1.92–3.45)	<0.001	1.59 (1.17–2.15)	0.003	1.59 (1.15–2.19)	0.005
Group3	56,841	47	0.19 (0.14–0.25)	2.42 (1.80–3.25)	<0.001	2.63 (1.96–3.54)	<0.001	1.72 (1.26–2.34)	0.001	1.71 (1.25–2.35)	0.001
Group4	1,891,461	644	0.08 (0.07–0.08)	REF		REF		REF		REF	
**Stroke**											
Group1	79,645	2,122	6.07 (5.82–6.34)	1.13 (1.08–1.18)	<0.001	1.13 (1.08–1.18)	<0.001	1.04 (0.99–1.08)	0.135	1.03 (0.99–1.08)	0.148
Group2	58,231	1,532	5.97 (5.68–6.27)	1.11 (1.05–1.17)	<0.001	1.13 (1.07–1.19)	<0.001	1.06 (1.01–1.12)	0.022	1.06 (1.01–1.12)	0.023

Group3	56,841	1,528	6.08 (5.78–6.39)	1.13 (1.07–1.19)	<0.001	1.17 (1.11–1.23)	<0.001	1.09 (1.03–1.15)	0.002	1.09 (1.03–1.15)	0.002
Group4	1,891,461	44,812	5.39 (5.34–5.44)	REF		REF		REF		REF	
**Heart Failure**											
Group1	79,645	1,206	3.45 (3.26–3.65)	1.92 (1.81–2.03)	<0.001	1.94 (1.83–2.05)	<0.001	1.43 (1.34–1.52)	<0.001	1.42 (1.34–1.52)	<0.001
Group2	58,231	679	2.64 (2.45–2.85)	1.47 (1.36–1.58)	<0.001	1.51 (1.40–1.64)	<0.001	1.22 (1.13–1.32)	<0.001	1.22 (1.13–1.32)	<0.001
Group3	56,841	713	2.84 (2.64–3.05)	1.57 (1.46–1.70)	<0.001	1.66 (1.54–1.79)	<0.001	1.33 (1.23–1.43)	<0.001	1.33 (1.23–1.43)	<0.001
Group4	1,891,461	15,012	1.80 (1.78–1.83)	REF		REF		REF		REF	
**Atrial fibrillation**											
Group1	79,645	720	2.06 (1.92–2.22)	1.12 (1.04–1.21)	0.003	1.14 (1.06–1.23)	<0.001	1.08 (1.00–1.16)	0.064	1.08 (0.99–1.16)	0.071
Group2	58,231	468	1.82 (1.66–2.00)	0.99 (0.90–1.08)	0.807	1.04 (0.95–1.14)	0.406	1.01 (0.92–1.11)	0.799	1.01 (0.92–1.11)	0.815
Group3	56,841	481	1.91 (1.75–2.09)	1.04 (0.95–1.14)	0.414	1.12 (1.02–1.23)	0.014	1.06 (0.96–1.16)	0.255	1.06 (0.96–1.16)	0.263
Group4	1,891,461	15,344	1.84 (1.82–1.87)	REF		REF		REF		REF	

Group1, Low-to-Low; Group2, Low-to-Normal; Group3, Normal-to-Low; Group4, Normal-to-Normal. Model 1 is unadjusted; Model 2 is adjusted for age and sex, as applicable; Model 3 is the fully adjusted multivariable model; Model 4 represents the Fine–Gray competing risk model adjusted for the same covariates as Model 3. Incidence rates are presented per 1,000 person-years with 95% confidence intervals. HDL-C, high-density lipoprotein cholesterol; HR, hazard ratio; sHR, subdistribution hazard ratio; CI, confidence interval.

Table S5. Association between HDL-C trajectory groups and cardiovascular disease risk, stratified by sex: female participants

				Model1		Model2		Model3		Model4	
	Person	Events	Incidence Rate per 1,000 Person Years (95% CI)	HR (95%CI)	P-value	HR (95%CI)	P-value	HR (95%CI)	P-value	sHR (95%CI)	P-value
**Composite CVD**											
Group1	5,035	218	10.93 (9.58–12.49)	1.46 (1.28–1.67)	<0.001	1.40 (1.23–1.60)	<0.001	1.12 (0.98–1.28)	0.109	1.12 (0.98–1.28)	0.109
Group2	6,045	222	9.54 (8.36–10.88)	1.27 (1.12–1.45)	<0.001	1.25 (1.10–1.43)	0.001	1.08 (0.94–1.23)	0.276	1.08 (0.94–1.23)	0.281
Group3	5,898	229	9.84 (8.65–11.20)	1.31 (1.15–1.50)	<0.001	1.33 (1.17–1.52)	<0.001	1.11 (0.97–1.27)	0.114	1.11 (0.97–1.27)	0.115
Group4	1,284,768	37,512	7.49 (7.42–7.57)	REF		REF		REF		REF	
**Myocardial Infarction**											
Group1	5,035	2	0.10 (0.03–0.36)	19.9 (4.72–84.2)	<0.001	16.9 (4.00–71.5)	<0.001	6.43 (1.28–32.4)	0.024	6.43 (1.16–35.6)	0.033
Group2	6,045	1	0.04 (0.01–0.24)	8.59 (1.16–63.4)	0.035	7.64 (1.03–56.5)	0.046	3.46 (0.45–26.7)	0.235	3.46 (0.40–30.2)	0.262
Group3	5,898	0	0.00 (0.00–0.16)	0.00 (0.00-.)	0.989	0.00 (0.00-.)	0.988	0.00 (0.00-.)	0.995	0.00 (0.00–0.00)	<0.001
Group4	1,284,768	25	0.00 (0.00–0.01)	REF		REF		REF		REF	
**Angina Pectoris**											
Group1	5,035	0	0.00 (0.00–0.19)	0.00 (0.00-.)	0.982	0.00 (0.00-.)	0.986	0.00 (0.00-.)	0.991	0.00 (0.00–0.00)	<0.001
Group2	6,045	1	0.04 (0.01–0.24)	5.80 (0.80–42.3)	0.083	5.26 (0.72–38.4)	0.101	2.33 (0.31–17.5)	0.410	2.33 (0.33–16.7)	0.398
Group3	5,898	1	0.04 (0.01–0.24)	5.81 (0.80–42.4)	0.082	5.80 (0.80–42.3)	0.083	2.16 (0.24–19.0)	0.489	2.16 (0.20–23.1)	0.526
Group4	1,284,768	37	0.01 (0.01–0.01)	REF		REF		REF		REF	
**Stroke**											
Group1	5,035	137	6.87 (5.81–8.12)	1.18 (0.99–1.39)	0.059	1.13 (0.96–1.34)	0.150	0.97 (0.82–1.15)	0.707	0.97 (0.81–1.15)	0.697

Group2	6,045	157	6.74 (5.77–7.89)	1.16 (0.99–1.35)	0.071	1.14 (0.97–1.33)	0.106	1.02 (0.87–1.20)	0.799	1.02 (0.87–1.19)	0.809
Group3	5,898	151	6.49 (5.54–7.61)	1.11 (0.95–1.30)	0.199	1.13 (0.96–1.32)	0.144	0.99 (0.84–1.17)	0.904	0.99 (0.84–1.17)	0.901
Group4	1,284,768	29,243	5.84 (5.77–5.91)	REF		REF		REF		REF	
**Heart Failure**											
Group1	5,035	51	2.56 (1.95–3.36)	2.67 (2.02–3.52)	<0.001	2.54 (1.93–3.34)	<0.001	1.44 (1.08–1.92)	0.013	1.44 (1.07–1.93)	0.015
Group2	6,045	42	1.80 (1.34–2.44)	1.89 (1.39–2.56)	<0.001	1.85 (1.36–2.50)	<0.001	1.29 (0.95–1.75)	0.101	1.29 (0.95–1.75)	0.101
Group3	5,898	51	2.19 (1.67–2.88)	2.29 (1.73–3.01)	<0.001	2.33 (1.77–3.07)	<0.001	1.46 (1.10–1.95)	0.009	1.46 (1.10–1.96)	0.010
Group4	1,284,768	4,794	0.96 (0.93–0.99)	REF		REF		REF		REF	
**Atrial fibrillation**											
Group1	5,035	28	1.40 (0.97–2.03)	2.06 (1.42–2.99)	<0.001	1.92 (1.32–2.78)	0.001	1.62 (1.10–2.37)	0.014	1.61 (1.10–2.36)	0.014
Group2	6,045	21	0.90 (0.59–1.38)	1.32 (0.86–2.03)	0.200	1.28 (0.83–1.96)	0.265	1.13 (0.73–1.74)	0.584	1.13 (0.73–1.74)	0.588
Group3	5,898	26	1.12 (0.76–1.64)	1.64 (1.11–2.41)	0.012	1.67 (1.14–2.46)	0.009	1.48 (1.00–2.20)	0.051	1.48 (1.00–2.19)	0.050
Group4	1,284,768	3,413	0.68 (0.66–0.71)	REF		REF		REF		REF	

Group1, Low-to-Low; Group2, Low-to-Normal; Group3, Normal-to-Low; Group4, Normal-to-Normal. Model 1 is unadjusted; Model 2 is adjusted for age and sex, as applicable; Model 3 is the fully adjusted multivariable model; Model 4 represents the Fine–Gray competing risk model adjusted for the same covariates as Model 3. Incidence rates are presented per 1,000 person-years with 95% confidence intervals. HDL-C, high-density lipoprotein cholesterol; HR, hazard ratio; sHR, subdistribution hazard ratio; CI, confidence interval.

Table S6. Association between HDL-C trajectory groups and cardiovascular disease risk, stratified by age: participants aged <40 years

				Model1		Model2		Model3		Model4	
	Person	Events	Incidence Rate per 1,000 Person Years (95% CI)	HR (95%CI)	P-value	HR (95%CI)	P-value	HR (95%CI)	P-value	sHR (95%CI)	P-value
**Composite CVD**											
Group1	22,658	517	5.75 (5.28–6.27)	1.47 (1.34–1.60)	<0.001	1.41 (1.29–1.54)	<0.001	1.18 (1.07–1.29)	0.001	1.18 (1.07–1.29)	0.001
Group2	18,464	349	4.77 (4.30–5.30)	1.22 (1.10–1.36)	<0.001	1.19 (1.07–1.33)	0.001	1.05 (0.94–1.17)	0.353	1.05 (0.94–1.17)	0.357
Group3	19,547	380	4.92 (4.45–5.44)	1.26 (1.14–1.39)	<0.001	1.25 (1.13–1.38)	<0.001	1.11 (0.99–1.23)	0.062	1.11 (0.99–1.23)	0.063
Group4	932,038	14,044	3.91 (3.85–3.98)	REF		REF		REF		REF	
**Myocardial Infarction**											
Group1	22,658	6	0.07 (0.03–0.15)	30.0 (10.4–86.6)	<0.001	17.0 (5.89–49.1)	<0.001	9.68 (2.98–31.4)	<0.001	9.65 (3.09–30.1)	<0.001
Group2	18,464	1	0.01 (0.00–0.08)	6.15 (0.77–49.2)	0.087	3.81 (0.48–30.5)	0.207	2.23 (0.27–18.4)	0.457	2.23 (0.26–19.5)	0.468
Group3	19,547	4	0.05 (0.02–0.13)	23.4 (7.04–77.6)	<0.001	15.6 (4.68–51.7)	<0.001	9.59 (2.56–35.9)	0.001	9.55 (2.10–43.5)	0.004
Group4	932,038	8	0.00 (0.00–0.00)	REF		REF		REF		REF	
**Angina Pectoris**											
Group1	22,658	4	0.04 (0.02–0.11)	13.3 (4.27–41.1)	<0.001	7.58 (2.44–23.6)	<0.001	3.45 (0.97–12.3)	0.056	3.45 (1.18–10.1)	0.023
Group2	18,464	1	0.01 (0.00–0.08)	4.08 (0.53–31.4)	0.177	2.54 (0.33–19.5)	0.371	1.07 (0.13–8.50)	0.951	1.07 (0.13–8.77)	0.950
Group3	19,547	1	0.01 (0.00–0.07)	3.84 (0.50–29.5)	0.196	2.57 (0.33–19.8)	0.364	1.29 (0.16–10.6)	0.816	1.28 (0.18–8.87)	0.805
Group4	932,038	12	0.00 (0.00–0.01)	REF		REF		REF		REF	
**Stroke**											
Group1	22,658	316	3.51 (3.15–3.92)	1.18 (1.05–1.32)	0.004	1.18 (1.05–1.32)	0.004	1.03 (0.92–1.16)	0.620	1.03 (0.92–1.16)	0.620

Group2	18,464	250	3.42 (3.02–3.87)	1.15 (1.01–1.30)	0.032	1.16 (1.02–1.32)	0.021	1.06 (0.93–1.20)	0.384	1.06 (0.93–1.20)	0.385
Group3	19,547	264	3.42 (3.03–3.86)	1.15 (1.02–1.30)	0.027	1.18 (1.04–1.33)	0.010	1.07 (0.94–1.22)	0.286	1.07 (0.94–1.22)	0.290
Group4	932,038	10,690	2.98 (2.92–3.03)	REF		REF		REF		REF	
**Heart Failure**											
Group1	22,658	128	1.42 (1.20–1.69)	2.42 (2.02–2.89)	<0.001	2.21 (1.84–2.65)	<0.001	1.49 (1.23–1.81)	<0.001	1.49 (1.22–1.81)	<0.001
Group2	18,464	62	0.85 (0.66–1.09)	1.44 (1.12–1.86)	0.005	1.35 (1.05–1.74)	0.021	1.02 (0.79–1.32)	0.867	1.02 (0.79–1.32)	0.869
Group3	19,547	76	0.98 (0.79–1.23)	1.68 (1.33–2.11)	<0.001	1.59 (1.26–2.00)	<0.001	1.22 (0.96–1.55)	0.097	1.22 (0.97–1.55)	0.095
Group4	932,038	2,106	0.59 (0.56–0.61)	REF		REF		REF		REF	
**Atrial fibrillation**											
Group1	22,658	63	0.70 (0.55–0.90)	2.05 (1.59–2.64)	<0.001	1.59 (1.23–2.05)	<0.001	1.44 (1.09–1.89)	0.009	1.44 (1.08–1.91)	0.012
Group2	18,464	35	0.48 (0.34–0.67)	1.40 (1.00–1.96)	0.049	1.14 (0.81–1.59)	0.453	1.06 (0.75–1.49)	0.738	1.06 (0.75–1.49)	0.742
Group3	19,547	35	0.45 (0.33–0.63)	1.33 (0.95–1.86)	0.095	1.11 (0.79–1.56)	0.535	1.06 (0.75–1.49)	0.759	1.06 (0.75–1.49)	0.757
Group4	932,038	1,228	0.34 (0.32–0.36)	REF		REF		REF		REF	

Group1, Low-to-Low; Group2, Low-to-Normal; Group3, Normal-to-Low; Group4, Normal-to-Normal. Model 1 is unadjusted; Model 2 is adjusted for age and sex, as applicable; Model 3 is the fully adjusted multivariable model; Model 4 represents the Fine–Gray competing risk model adjusted for the same covariates as Model 3. Incidence rates are presented per 1,000 person-years with 95% confidence intervals. HDL-C, high-density lipoprotein cholesterol; HR, hazard ratio; sHR, subdistribution hazard ratio; CI, confidence interval.

Table S7. Association between HDL-C trajectory groups and cardiovascular disease risk, stratified by age: participants aged 40–64 years

				Model1		Model2		Model3		Model4	
	Person	Events	Incidence Rate per 1,000 Person Years (95% CI)	HR (95%CI)	P-value	HR (95%CI)	P-value	HR (95%CI)	P-value	sHR (95%CI)	P-value
**Composite CVD**											
Group1	59,814	3,678	13.50 (13.07–13.94)	1.38 (1.34–1.43)	<0.001	1.33 (1.29–1.38)	<0.001	1.16 (1.12–1.20)	<0.001	1.16 (1.12–1.20)	<0.001
Group2	44,271	2,465	12.20 (11.73–12.69)	1.25 (1.20–1.30)	<0.001	1.21 (1.16–1.26)	<0.001	1.10 (1.06–1.14)	<0.001	1.10 (1.05–1.14)	<0.001
Group3	41,755	2,493	12.93 (12.43–13.45)	1.32 (1.27–1.38)	<0.001	1.29 (1.24–1.34)	<0.001	1.15 (1.10–1.20)	<0.001	1.15 (1.10–1.20)	<0.001
Group4	2,152,179	92,153	9.75 (9.69–9.81)	REF		REF		REF		REF	
**Myocardial Infarction**											
Group1	59,814	60	0.22 (0.17–0.28)	5.45 (4.15–7.16)	<0.001	3.98 (3.03–5.23)	<0.001	2.11 (1.57–2.83)	<0.001	2.10 (1.54–2.87)	<0.001
Group2	44,271	28	0.14 (0.10–0.20)	3.42 (2.33–5.02)	<0.001	2.57 (1.75–3.77)	<0.001	1.50 (1.01–2.22)	0.045	1.50 (0.99–2.26)	0.055
Group3	41,755	26	0.13 (0.09–0.20)	3.32 (2.23–4.95)	<0.001	2.51 (1.69–3.74)	<0.001	1.55 (1.03–2.35)	0.036	1.55 (1.04–2.31)	0.031
Group4	2,152,179	381	0.04 (0.04–0.04)	REF		REF		REF		REF	
**Angina Pectoris**											
Group1	59,814	76	0.28 (0.22–0.35)	4.30 (3.38–5.45)	<0.001	3.18 (2.50–4.04)	<0.001	1.84 (1.42–2.38)	<0.001	1.84 (1.42–2.38)	<0.001
Group2	44,271	46	0.23 (0.17–0.30)	3.51 (2.60–4.74)	<0.001	2.67 (1.98–3.60)	<0.001	1.65 (1.21–2.25)	0.002	1.65 (1.19–2.29)	0.003
Group3	41,755	45	0.23 (0.17–0.31)	3.59 (2.65–4.86)	<0.001	2.75 (2.03–3.73)	<0.001	1.80 (1.31–2.47)	<0.001	1.80 (1.30–2.50)	<0.001
Group4	2,152,179	611	0.06 (0.06–0.07)	REF		REF		REF		REF	
**Stroke**											
Group1	59,814	1,855	6.81 (6.50–7.12)	1.08 (1.04–1.14)	0.001	1.14 (1.09–1.19)	<0.001	1.04 (0.99–1.09)	0.115	1.04 (0.99–1.09)	0.125

Group2	44,271	1,357	6.72 (6.37–7.08)	1.07 (1.01–1.13)	0.014	1.12 (1.06–1.18)	<0.001	1.05 (1.00–1.11)	0.073	1.05 (0.99–1.11)	0.077
Group3	41,755	1,352	7.01 (6.65–7.40)	1.12 (1.06–1.18)	<0.001	1.17 (1.11–1.24)	<0.001	1.09 (1.03–1.15)	0.004	1.08 (1.03–1.15)	0.004
Group4	2,152,179	59,257	6.27 (6.22–6.32)	REF		REF		REF		REF	
**Heart Failure**											
Group1	59,814	1,058	3.88 (3.66–4.12)	2.25 (2.11–2.39)	<0.001	1.96 (1.84–2.08)	<0.001	1.42 (1.33–1.51)	<0.001	1.41 (1.32–1.51)	<0.001
Group2	44,271	627	3.10 (2.87–3.36)	1.79 (1.65–1.94)	<0.001	1.58 (1.46–1.71)	<0.001	1.27 (1.17–1.37)	<0.001	1.26 (1.17–1.37)	<0.001
Group3	41,755	649	3.37 (3.12–3.64)	1.94 (1.79–2.10)	<0.001	1.72 (1.59–1.86)	<0.001	1.35 (1.24–1.47)	<0.001	1.35 (1.24–1.47)	<0.001
Group4	2,152,179	16,257	1.72 (1.69–1.75)	REF		REF		REF		REF	
**Atrial fibrillation**											
Group1	59,814	629	2.31 (2.13–2.50)	1.40 (1.29–1.51)	<0.001	1.15 (1.06–1.25)	0.001	1.07 (0.98–1.16)	0.108	1.07 (0.98–1.16)	0.118
Group2	44,271	407	2.01 (1.83–2.22)	1.21 (1.10–1.34)	<0.001	1.02 (0.93–1.13)	0.657	0.99 (0.89–1.09)	0.809	0.99 (0.89–1.09)	0.794
Group3	41,755	421	2.18 (1.98–2.40)	1.32 (1.20–1.45)	<0.001	1.12 (1.01–1.23)	0.027	1.04 (0.94–1.15)	0.409	1.04 (0.94–1.15)	0.415
Group4	2,152,179	15,647	1.66 (1.63–1.68)	REF		REF		REF		REF	

Group1, Low-to-Low; Group2, Low-to-Normal; Group3, Normal-to-Low; Group4, Normal-to-Normal. Model 1 is unadjusted; Model 2 is adjusted for age and sex, as applicable; Model 3 is the fully adjusted multivariable model; Model 4 represents the Fine–Gray competing risk model adjusted for the same covariates as Model 3. Incidence rates are presented per 1,000 person-years with 95% confidence intervals. HDL-C, high-density lipoprotein cholesterol; HR, hazard ratio; sHR, subdistribution hazard ratio; CI, confidence interval.

Table S8. Association between HDL-C trajectory groups and cardiovascular disease risk, stratified by age: participants aged ≥65 years

				Model1		Model2		Model3		Model4	
	Person	Events	Incidence Rate per 1,000 Person Years (95% CI)	HR (95%CI)	P-value	HR (95%CI)	P-value	HR (95%CI)	P-value	sHR (95%CI)	P-value
**Composite CVD**											
Group1	2,208	220	31.74 (27.82–36.23)	1.19 (1.04–1.36)	0.010	1.09 (0.95–1.25)	0.215	0.99 (0.86–1.14)	0.914	0.99 (0.86–1.14)	0.902
Group2	1,541	164	33.86 (29.07–39.46)	1.27 (1.09–1.48)	0.003	1.19 (1.02–1.39)	0.027	1.12 (0.96–1.31)	0.149	1.12 (0.96–1.31)	0.150
Group3	1,437	155	34.67 (29.63–40.58)	1.30 (1.11–1.52)	0.001	1.20 (1.02–1.40)	0.027	1.09 (0.93–1.28)	0.297	1.09 (0.92–1.28)	0.306
Group4	92,012	7,528	26.59 (25.99–27.20)	REF		REF		REF		REF	
**Myocardial Infarction**											
Group1	2,208	3	0.43 (0.16–1.27)	3.30 (1.02–10.7)	0.047	2.60 (0.80–8.48)	0.114	1.86 (0.53–6.57)	0.336	1.86 (0.57–6.08)	0.305
Group2	1,541	1	0.21 (0.05–1.15)	1.59 (0.22–11.6)	0.647	1.32 (0.18–9.65)	0.784	0.83 (0.11–6.26)	0.860	0.84 (0.10–6.97)	0.869
Group3	1,437	0	0.00 (0.01–0.83)	0.00 (0.00-0.00)	0.983	0.00 (0.00-0.00)	0.983	0.00 (0.00-0.00)	0.989	0.00 (0.00–0.00)	<0.001
Group4	92,012	37	0.13 (0.09–0.18)	REF		REF		REF		REF	
**Angina Pectoris**											
Group1	2,208	2	0.29 (0.09–1.04)	1.41 (0.34–5.78)	0.631	1.03 (0.25–4.23)	0.966	0.65 (0.15–2.77)	0.561	0.65 (0.16–2.66)	0.549
Group2	1,541	2	0.41 (0.13–1.49)	1.99 (0.49–8.17)	0.337	1.56 (0.38–6.38)	0.539	1.07 (0.26–4.45)	0.925	1.07 (0.26–4.44)	0.922
Group3	1,437	2	0.45 (0.14–1.62)	2.17 (0.53–8.89)	0.281	1.62 (0.40–6.66)	0.500	1.02 (0.23–4.50)	0.982	1.02 (0.23–4.59)	0.983
Group4	92,012	58	0.20 (0.16–0.26)	REF		REF		REF		REF	
**Stroke**											
Group1	2,208	88	12.70 (10.31–15.64)	0.87 (0.71–1.08)	0.212	0.87 (0.71–1.08)	0.211	0.80 (0.64–1.00)	0.047	0.80 (0.64–1.00)	0.046

Group2	1,541	82	16.93 (13.65–21.01)	1.16 (0.93–1.45)	0.177	1.17 (0.94–1.45)	0.164	1.11 (0.89–1.38)	0.353	1.11 (0.89–1.38)	0.354
Group3	1,437	63	14.09 (11.02–18.03)	0.97 (0.75–1.24)	0.792	0.97 (0.75–1.24)	0.789	0.88 (0.68–1.14)	0.340	0.88 (0.68–1.14)	0.336
Group4	92,012	4,108	14.51 (14.07–14.96)	REF		REF		REF		REF	
**Heart Failure**											
Group1	2,208	71	10.24 (8.13–12.92)	2.00 (1.58–2.54)	<0.001	1.74 (1.37–2.21)	<0.001	1.41 (1.10–1.81)	0.007	1.41 (1.10–1.81)	0.007
Group2	1,541	32	6.61 (4.69–9.33)	1.28 (0.90–1.82)	0.162	1.16 (0.82–1.65)	0.410	1.00 (0.70–1.42)	0.991	1.00 (0.70–1.42)	1.000
Group3	1,437	39	8.72 (6.39–11.93)	1.69 (1.23–2.33)	0.001	1.49 (1.08–2.05)	0.014	1.23 (0.88–1.70)	0.224	1.22 (0.88–1.69)	0.234
Group4	92,012	1,443	5.10 (4.84–5.37)	REF		REF		REF		REF	
**Atrial fibrillation**											
Group1	2,208	56	8.08 (6.23–10.49)	1.22 (0.93–1.59)	0.147	0.98 (0.75–1.28)	0.868	0.98 (0.74–1.29)	0.889	0.98 (0.75–1.28)	0.878
Group2	1,541	47	9.70 (7.31–12.90)	1.46 (1.09–1.95)	0.010	1.24 (0.93–1.66)	0.147	1.25 (0.93–1.68)	0.132	1.25 (0.93–1.68)	0.136
Group3	1,437	51	11.41 (8.69–15.00)	1.72 (1.30–2.27)	<0.001	1.41 (1.07–1.86)	0.016	1.40 (1.05–1.86)	0.022	1.39 (1.05–1.85)	0.023
Group4	92,012	1,882	6.65 (6.35–6.95)	REF		REF		REF		REF	

Group1, Low-to-Low; Group2, Low-to-Normal; Group3, Normal-to-Low; Group4, Normal-to-Normal. Model 1 is unadjusted; Model 2 is adjusted for age and sex, as applicable; Model 3 is the fully adjusted multivariable model; Model 4 represents the Fine–Gray competing risk model adjusted for the same covariates as Model 3. Incidence rates are presented per 1,000 person-years with 95% confidence intervals. HDL-C, high-density lipoprotein cholesterol; HR, hazard ratio; sHR, subdistribution hazard ratio; CI, confidence interval.

Table S9. Association between HDL-C trajectory groups and cardiovascular disease risk among participants using lipid-lowering medications

	Person	Events	Incidence Rate per 1,000 Person Years (95% CI)	Model1 HR (95%CI)	Model1 P-value	Model2 HR (95%CI)	Model2 P-value	Model3 HR (95%CI)	Model3 P-value	Model4 sHR (95%CI)	Model4 P-value
**Composite CVD**											
Group1	9714	796	20.22 (18.87–21.68)	1.21 (1.12–1.30)	<0.001	1.30 (1.21–1.39)	<0.001	1.17 (1.08–1.26)	<0.001	1.17 (1.08–1.26)	<0.001
Group2	7415	552	18.61 (17.13–20.23)	1.11 (1.02–1.21)	0.015	1.19 (1.09–1.30)	<0.001	1.12 (1.03–1.22)	0.009	1.12 (1.03–1.22)	0.009
Group3	6128	531	20.71 (19.02–22.55)	1.24 (1.13–1.35)	<0.001	1.30 (1.20–1.42)	<0.001	1.21 (1.10–1.32)	<0.001	1.20 (1.10–1.32)	<0.001
Group4	211991	13713	16.75 (16.47–17.03)	REF		REF		REF		REF	
**Myocardial Infarction**											
Group1	9714	6	0.15 (0.07–0.33)	1.40 (0.61–3.19)	0.428	1.19 (0.52–2.73)	0.679	0.88 (0.37–2.09)	0.778	0.88 (0.36–2.16)	0.784
Group2	7415	7	0.24 (0.12–0.49)	2.16 (1.00–4.67)	0.049	1.89 (0.87–4.08)	0.107	1.47 (0.67–3.24)	0.335	1.47 (0.66–3.29)	0.346
Group3	6128	8	0.31 (0.16–0.61)	2.84 (1.38–5.86)	0.005	2.44 (1.18–5.04)	0.016	2.06 (0.97–4.36)	0.061	2.05 (0.99–4.25)	0.053
Group4	211991	89	0.11 (0.09–0.13)	REF		REF		REF		REF	
**Angina Pectoris**											
Group1	9714	19	0.48 (0.31–0.75)	2.61 (1.62–4.21)	<0.001	2.34 (1.45–3.78)	0.001	1.75 (1.05–2.93)	0.033	1.75 (1.05–2.91)	0.031
Group2	7415	14	0.47 (0.28–0.79)	2.56 (1.48–4.43)	0.001	2.33 (1.35–4.04)	0.003	1.95 (1.11–3.42)	0.019	1.95 (1.10–3.47)	0.023
Group3	6128	12	0.47 (0.27–0.82)	2.51 (1.39–4.52)	0.002	2.24 (1.24–4.05)	0.007	1.82 (0.99–3.35)	0.055	1.82 (0.97–3.41)	0.064
Group4	211991	150	0.18 (0.16–0.21)	REF		REF		REF		REF	
**Stroke**											
Group1	9714	352	8.94 (8.06–9.93)	0.94 (0.84–1.05)	0.258	1.08 (0.97–1.20)	0.180	1.04 (0.93–1.17)	0.474	1.04 (0.93–1.16)	0.484

Group2	7415	262	8.84 (7.83–9.97)	0.93 (0.82–1.05)	0.241	1.05 (0.93–1.19)	0.428	1.03 (0.91–1.17)	0.624	1.03 (0.91–1.17)	0.629
Group3	6128	239	9.32 (8.21–10.58)	0.98 (0.86–1.11)	0.760	1.10 (0.96–1.25)	0.159	1.06 (0.93–1.21)	0.390	1.06 (0.93–1.21)	0.397
Group4	211991	7787	9.51 (9.30–9.72)	REF		REF		REF		REF	
**Heart Failure**											
Group1	9714	273	6.94 (6.16–7.81)	1.89 (1.67–2.14)	<0.001	1.87 (1.65–2.12)	<0.001	1.42 (1.24–1.62)	<0.001	1.42 (1.24–1.62)	<0.001
Group2	7415	160	5.40 (4.62–6.30)	1.48 (1.26–1.73)	<0.001	1.47 (1.25–1.72)	<0.001	1.24 (1.06–1.46)	0.008	1.25 (1.06–1.46)	0.007
Group3	6128	177	6.90 (5.96–8.00)	1.88 (1.61–2.18)	<0.001	1.84 (1.58–2.14)	<0.001	1.53 (1.31–1.79)	<0.001	1.53 (1.30–1.79)	<0.001
Group4	211991	2983	3.64 (3.51–3.78)	REF		REF		REF		REF	
**Atrial fibrillation**											
Group1	9714	146	3.71 (3.16–4.36)	1.13 (0.96–1.33)	0.155	1.14 (0.97–1.35)	0.113	1.06 (0.89–1.26)	0.530	1.06 (0.89–1.26)	0.537
Group2	7415	109	3.68 (3.05–4.43)	1.12 (0.92–1.35)	0.259	1.14 (0.94–1.39)	0.170	1.11 (0.92–1.35)	0.273	1.11 (0.92–1.35)	0.273
Group3	6128	95	3.70 (3.03–4.53)	1.13 (0.92–1.39)	0.244	1.13 (0.92–1.38)	0.256	1.05 (0.85–1.30)	0.624	1.05 (0.85–1.30)	0.635
Group4	211991	2704	3.30 (3.18–3.43)	REF		REF		REF		REF	

Group1, Low-to-Low; Group2, Low-to-Normal; Group3, Normal-to-Low; Group4, Normal-to-Normal. Model 1 is unadjusted; Model 2 is adjusted for age and sex, as applicable; Model 3 is the fully adjusted multivariable model; Model 4 represents the Fine–Gray competing risk model adjusted for the same covariates as Model 3. Incidence rates are presented per 1,000 person-years with 95% confidence intervals. HDL-C, high-density lipoprotein cholesterol; HR, hazard ratio; sHR, subdistribution hazard ratio; CI, confidence interval.

Table S10. Association between HDL-C trajectory groups and cardiovascular disease risk among participants using lipid-lowering medications, stratified by sex: male participants

				Model1		Model2		Model3		Model4	
	Person	Events	Incidence Rate per 1,000 Person Years (95% CI)	HR (95%CI)	P-value	HR (95%CI)	P-value	HR (95%CI)	P-value	sHR (95%CI)	P-value
**Composite CVD**											
Group1	9124	753	20.26 (18.87–21.76)	1.16 (1.08–1.25)	<0.001	1.29 (1.20–1.39)	<0.001	1.15 (1.06–1.24)	<0.001	1.15 (1.06–1.24)	<0.001
Group2	6716	510	18.77 (17.21–20.47)	1.07 (0.98–1.17)	0.115	1.19 (1.09–1.30)	<0.001	1.12 (1.02–1.22)	0.015	1.12 (1.02–1.22)	0.016
Group3	5555	496	21.07 (19.29–23.01)	1.21 (1.10–1.32)	<0.001	1.31 (1.20–1.44)	<0.001	1.21 (1.10–1.33)	<0.001	1.21 (1.10–1.33)	<0.001
Group4	141620	9963	17.47 (17.13–17.82)	REF		REF		REF		REF	
**Myocardial Infarction**											
Group1	9124	6	0.16 (0.08–0.35)	1.08 (0.47–2.48)	0.849	1.20 (0.52–2.75)	0.667	0.93 (0.39–2.19)	0.861	0.92 (0.38–2.26)	0.864
Group2	6716	7	0.26 (0.13–0.53)	1.73 (0.80–3.74)	0.164	1.90 (0.88–4.12)	0.102	1.51 (0.69–3.32)	0.306	1.51 (0.67–3.39)	0.319
Group3	5555	8	0.34 (0.17–0.67)	2.27 (1.10–4.69)	0.027	2.46 (1.19–5.09)	0.015	2.15 (1.01–4.56)	0.047	2.15 (1.04–4.43)	0.039
Group4	141620	85	0.15 (0.12–0.18)	REF		REF		REF		REF	
**Angina Pectoris**											
Group1	9124	19	0.51 (0.33–0.80)	2.08 (1.29–3.36)	0.003	2.37 (1.47–3.84)	<0.001	1.77 (1.06–2.97)	0.029	1.77 (1.06–2.95)	0.028
Group2	6716	14	0.52 (0.31–0.86)	2.10 (1.21–3.64)	0.008	2.38 (1.37–4.12)	0.002	2.01 (1.15–3.53)	0.014	2.01 (1.14–3.57)	0.017
Group3	5555	12	0.51 (0.29–0.89)	2.06 (1.14–3.71)	0.016	2.29 (1.27–4.13)	0.006	1.86 (1.01–3.42)	0.048	1.86 (0.99–3.49)	0.055
Group4	141620	140	0.25 (0.21–0.29)	REF		REF		REF		REF	
**Stroke**											
Group1	9124	322	8.66 (7.77–9.66)	0.97 (0.87–1.09)	0.590	1.05 (0.94–1.18)	0.354	1.01 (0.90–1.14)	0.834	1.01 (0.90–1.14)	0.848

Group2	6716	237	8.72 (7.68–9.90)	0.98 (0.86–1.11)	0.716	1.06 (0.93–1.20)	0.407	1.03 (0.91–1.18)	0.621	1.03 (0.91–1.18)	0.624
Group3	5555	217	9.22 (8.07–10.53)	1.03 (0.90–1.18)	0.652	1.10 (0.96–1.27)	0.153	1.06 (0.92–1.22)	0.399	1.06 (0.92–1.22)	0.410
Group4	141620	5096	8.94 (8.69–9.18)	REF		REF		REF		REF	
**Heart Failure**											
Group1	9124	265	7.13 (6.32–8.04)	1.70 (1.50–1.93)	<0.001	1.88 (1.65–2.13)	<0.001	1.43 (1.25–1.64)	<0.001	1.43 (1.25–1.64)	<0.001
Group2	6716	149	5.48 (4.67–6.44)	1.31 (1.11–1.54)	0.002	1.44 (1.22–1.69)	<0.001	1.23 (1.04–1.45)	0.016	1.23 (1.04–1.45)	0.016
Group3	5555	171	7.26 (6.25–8.44)	1.72 (1.48–2.01)	<0.001	1.87 (1.60–2.18)	<0.001	1.57 (1.34–1.84)	<0.001	1.57 (1.33–1.85)	<0.001
Group4	141620	2392	4.19 (4.03–4.37)	REF		REF		REF		REF	
**Atrial fibrillation**											
Group1	9124	141	3.79 (3.22–4.47)	0.96 (0.81–1.14)	0.668	1.13 (0.95–1.34)	0.156	1.05 (0.88–1.25)	0.621	1.04 (0.87–1.25)	0.629
Group2	6716	103	3.79 (3.13–4.60)	0.96 (0.79–1.17)	0.698	1.12 (0.92–1.37)	0.251	1.10 (0.90–1.34)	0.370	1.10 (0.90–1.34)	0.370
Group3	5555	88	3.74 (3.04–4.61)	0.95 (0.77–1.18)	0.659	1.08 (0.88–1.34)	0.462	1.01 (0.82–1.26)	0.896	1.01 (0.81–1.26)	0.905
Group4	141620	2250	3.95 (3.79–4.11)	REF		REF		REF		REF	

Group1, Low-to-Low; Group2, Low-to-Normal; Group3, Normal-to-Low; Group4, Normal-to-Normal. Model 1 is unadjusted; Model 2 is adjusted for age and sex, as applicable; Model 3 is the fully adjusted multivariable model; Model 4 represents the Fine–Gray competing risk model adjusted for the same covariates as Model 3. Incidence rates are presented per 1,000 person-years with 95% confidence intervals. HDL-C, high-density lipoprotein cholesterol; HR, hazard ratio; sHR, subdistribution hazard ratio; CI, confidence interval.

Table S11. Association between HDL-C trajectory groups and cardiovascular disease risk among participants using lipid-lowering medications, stratified by sex: female participants

				Model1		Model2		Model3		Model4	
	Person	Events	Incidence Rate per 1,000 Person Years (95% CI)	HR (95%CI)	P-value	HR (95%CI)	P-value	HR (95%CI)	P-value	sHR (95%CI)	P-value
**Composite CVD**											
Group1	590	43	19.60 (14.57–26.40)	1.30 (0.96–1.75)	0.089	1.50 (1.11–2.02)	0.009	1.32 (0.97–1.80)	0.080	1.32 (0.97–1.78)	0.075
Group2	699	42	16.95 (12.56–22.92)	1.12 (0.83–1.52)	0.454	1.25 (0.92–1.70)	0.148	1.16 (0.85–1.57)	0.344	1.16 (0.85–1.58)	0.352
Group3	573	35	16.67 (12.01–23.19)	1.10 (0.79–1.54)	0.560	1.21 (0.87–1.69)	0.253	1.09 (0.78–1.53)	0.609	1.09 (0.78–1.53)	0.598
Group4	70371	3750	15.09 (14.61–15.58)	REF		REF		REF		REF	
**Myocardial Infarction**											
Group1	590	0	0.00 (0.01–1.68)	0.00 (0.00–.)	0.997	0.00 (0.00–.)	0.997	0.00 (0.00–.)	0.998	0.00 (0.00–0.00)	<0.001
Group2	699	0	0.00 (0.01–1.49)	0.00 (0.00–.)	0.997	0.00 (0.00–.)	0.997	0.00 (0.00–.)	0.999	0.00 (0.00–0.00)	<0.001
Group3	573	0	0.00 (0.01–1.76)	0.00 (0.00–.)	0.997	0.00 (0.00–.)	0.997	0.00 (0.00–.)	0.996	0.00 (0.00–0.00)	<0.001
Group4	70371	4	0.02 (0.01–0.04)	REF		REF		REF		REF	
**Angina Pectoris**											
Group1	590	0	0.00 (0.01–1.68)	0.00 (0.00–.)	0.995	0.00 (0.00–.)	0.995	0.00 (0.00–.)	0.998	0.00 (0.00–0.00)	<0.001
Group2	699	0	0.00 (0.01–1.49)	0.00 (0.00–.)	0.995	0.00 (0.00–.)	0.995	0.00 (0.00–.)	0.998	0.00 (0.00–0.00)	<0.001
Group3	573	0	0.00 (0.01–1.76)	0.00 (0.00–.)	0.996	0.00 (0.00–.)	0.996	0.00 (0.00–.)	0.998	0.00 (0.00–0.00)	<0.001
Group4	70371	10	0.04 (0.02–0.07)	REF		REF		REF		REF	
**Stroke**											
Group1	590	30	13.68 (9.60–19.52)	1.26 (0.88–1.81)	0.205	1.42 (0.99–2.04)	0.056	1.37 (0.95–1.98)	0.096	1.37 (0.95–1.96)	0.089

Group2	699	25	10.09 (6.86–14.90)	0.93 (0.63–1.38)	0.722	1.02 (0.69–1.51)	0.919	1.00 (0.67–1.48)	0.997	1.00 (0.67–1.49)	0.994
Group3	573	22	10.48 (6.95–15.87)	0.97 (0.64–1.47)	0.873	1.05 (0.69–1.59)	0.828	1.01 (0.66–1.55)	0.947	1.02 (0.67–1.54)	0.943
Group4	70371	2691	10.83 (10.43–11.24)	REF		REF		REF		REF	
**Heart Failure**											
Group1	590	8	3.65 (1.88–7.19)	1.52 (0.76–3.06)	0.236	1.76 (0.88–3.55)	0.111	1.08 (0.52–2.21)	0.844	1.07 (0.52–2.23)	0.848
Group2	699	11	4.44 (2.50–7.94)	1.87 (1.03–3.40)	0.039	2.10 (1.15–3.81)	0.015	1.57 (0.86–2.87)	0.140	1.57 (0.86–2.88)	0.145
Group3	573	6	2.86 (1.34–6.22)	1.20 (0.54–2.68)	0.655	1.33 (0.59–2.97)	0.490	0.85 (0.37–1.97)	0.713	0.86 (0.36–2.02)	0.724
Group4	70371	591	2.38 (2.19–2.58)	REF		REF		REF		REF	
**Atrial fibrillation**											
Group1	590	5	2.28 (1.00–5.32)	1.26 (0.52–3.03)	0.613	1.61 (0.66–3.88)	0.293	1.51 (0.61–3.73)	0.368	1.51 (0.62–3.67)	0.367
Group2	699	6	2.42 (1.14–5.27)	1.32 (0.59–2.96)	0.495	1.60 (0.71–3.58)	0.253	1.53 (0.68–3.44)	0.307	1.53 (0.67–3.45)	0.310
Group3	573	7	3.33 (1.65–6.87)	1.83 (0.87–3.86)	0.112	2.14 (1.01–4.51)	0.046	2.03 (0.94–4.37)	0.071	2.03 (0.95–4.35)	0.069
Group4	70371	454	1.83 (1.67–2.00)	REF		REF		REF		REF	

Group1, Low-to-Low; Group2, Low-to-Normal; Group3, Normal-to-Low; Group4, Normal-to-Normal. Model 1 is unadjusted; Model 2 is adjusted for age and sex, as applicable; Model 3 is the fully adjusted multivariable model; Model 4 represents the Fine–Gray competing risk model adjusted for the same covariates as Model 3. Incidence rates are presented per 1,000 person-years with 95% confidence intervals. HDL-C, high-density lipoprotein cholesterol; HR, hazard ratio; sHR, subdistribution hazard ratio; CI, confidence interval.

Table S12. Association between HDL-C trajectory groups and cardiovascular disease risk among participants using lipid-lowering medications, stratified by age: participants aged <40 years

				Model1		Model2		Model3		Model4	
	Person	Events	Incidence Rate per 1,000 Person Years (95% CI)	HR (95%CI)	P-value	HR (95%CI)	P-value	HR (95%CI)	P-value	sHR (95%CI)	P-value
**Composite CVD**											
Group1	880	37	11.02 (8.01–15.19)	1.46 (1.03–2.06)	0.033	1.50 (1.06–2.12)	0.023	1.25 (0.85–1.83)	0.257	1.25 (0.85–1.84)	0.260
Group2	582	19	8.59 (5.53–13.42)	1.13 (0.71–1.81)	0.598	1.16 (0.72–1.85)	0.546	1.04 (0.65–1.67)	0.862	1.04 (0.65–1.67)	0.873
Group3	456	22	11.60 (7.69–17.57)	1.50 (0.97–2.31)	0.070	1.52 (0.98–2.36)	0.061	1.28 (0.81–2.02)	0.286	1.28 (0.81–2.02)	0.293
Group4	8462	249	7.63 (6.74–8.63)	REF		REF		REF		REF	
**Myocardial Infarction**											
Group1	880	0	0.00 (0.01–1.10)	.	.	.	.	.	.	.	.
Group2	582	0	0.00 (0.01–1.67)	.	.	.	.	.	.	.	.
Group3	456	0	0.00 (0.01–1.95)	.	.	.	.	.	.	.	.
Group4	8462	0	0.00 (0.00–0.11)	REF		REF		REF		REF	
**Angina Pectoris**											
Group1	880	1	0.30 (0.07–1.66)	5.07 (0.46–56.0)	0.185	4.33 (0.39–47.9)	0.232	63.6 (1.43–2818)	0.032	63.7 (0.38–11E3)	0.112
Group2	582	1	0.45 (0.11–2.52)	7.55 (0.68–83.3)	0.099	6.44 (0.58–71.4)	0.129	19.2 (0.72–516)	0.078	19.3 (6.83–54.3)	<0.001
Group3	456	0	0.00 (0.01–1.95)	0.00 (0.00–.)	0.995	0.00 (0.00–.)	0.998	0.00 (0.00–.)	1.000	0.00 (0.00–0.00)	<0.001
Group4	8462	2	0.06 (0.02–0.22)	REF		REF		REF		REF	
**Stroke**											
Group1	880	23	6.85 (4.58–10.28)	1.58 (1.02–2.46)	0.041	1.69 (1.08–2.63)	0.022	1.42 (0.87–2.32)	0.165	1.42 (0.88–2.28)	0.148

Group2	582	14	6.33 (3.80–10.62)	1.46 (0.84–2.53)	0.177	1.54 (0.88–2.67)	0.127	1.39 (0.80–2.43)	0.246	1.39 (0.80–2.42)	0.247
Group3	456	13	6.86 (4.04–11.72)	1.54 (0.87–2.71)	0.137	1.61 (0.91–2.85)	0.101	1.37 (0.76–2.48)	0.295	1.37 (0.76–2.47)	0.292
Group4	8462	143	4.38 (3.72–5.16)	REF		REF		REF		REF	
**Heart Failure**											
Group1	880	12	3.57 (2.06–6.24)	1.61 (0.87–2.96)	0.128	1.55 (0.84–2.86)	0.164	1.38 (0.70–2.71)	0.353	1.38 (0.69–2.76)	0.365
Group2	582	3	1.36 (0.49–3.97)	0.61 (0.19–1.93)	0.399	0.58 (0.18–1.86)	0.363	0.51 (0.16–1.63)	0.253	0.51 (0.16–1.65)	0.258
Group3	456	7	3.69 (1.82–7.61)	1.63 (0.75–3.55)	0.216	1.57 (0.72–3.42)	0.255	1.29 (0.57–2.90)	0.537	1.29 (0.57–2.93)	0.542
Group4	8462	73	2.24 (1.78–2.81)	REF		REF		REF		REF	
**Atrial fibrillation**											
Group1	880	1	0.30 (0.07–1.66)	0.31 (0.04–2.28)	0.250	0.32 (0.04–2.39)	0.270	0.17 (0.02–1.45)	0.105	0.17 (0.01–2.24)	0.177
Group2	582	1	0.45 (0.11–2.52)	0.48 (0.06–3.48)	0.464	0.49 (0.07–3.57)	0.477	0.43 (0.06–3.21)	0.411	0.43 (0.06–3.12)	0.403
Group3	456	2	1.05 (0.33–3.81)	1.10 (0.26–4.59)	0.899	1.12 (0.27–4.69)	0.879	0.77 (0.17–3.45)	0.730	0.77 (0.15–3.85)	0.747
Group4	8462	31	0.95 (0.67–1.35)	REF		REF		REF		REF	

Group1, Low-to-Low; Group2, Low-to-Normal; Group3, Normal-to-Low; Group4, Normal-to-Normal. Model 1 is unadjusted; Model 2 is adjusted for age and sex, as applicable; Model 3 is the fully adjusted multivariable model; Model 4 represents the Fine–Gray competing risk model adjusted for the same covariates as Model 3. Incidence rates are presented per 1,000 person-years with 95% confidence intervals. HDL-C, high-density lipoprotein cholesterol; HR, hazard ratio; sHR, subdistribution hazard ratio; CI, confidence interval.

Table S13. Association between HDL-C trajectory groups and cardiovascular disease risk among participants using lipid-lowering medications, stratified by age: participants aged 40–64 years

				Model1		Model2		Model3		Model4	
	Person	Events	Incidence Rate per 1,000 Person Years (95% CI)	HR (95%CI)	P-value	HR (95%CI)	P-value	HR (95%CI)	P-value	sHR (95%CI)	P-value
**Composite CVD**											
Group1	8258	687	20.02 (18.58–21.57)	1.26 (1.16–1.36)	<0.001	1.29 (1.19–1.39)	<0.001	1.15 (1.06–1.25)	0.001	1.15 (1.06–1.25)	0.001
Group2	6374	477	18.27 (16.70–19.98)	1.15 (1.05–1.26)	0.003	1.18 (1.07–1.29)	<0.001	1.11 (1.01–1.21)	0.032	1.11 (1.01–1.21)	0.033
Group3	5257	456	20.31 (18.53–22.26)	1.27 (1.16–1.40)	<0.001	1.30 (1.18–1.43)	<0.001	1.20 (1.09–1.32)	<0.001	1.20 (1.09–1.32)	<0.001
Group4	179617	11367	15.90 (15.61–16.19)	REF		REF		REF		REF	
**Myocardial Infarction**											
Group1	8258	6	0.17 (0.08–0.38)	1.62 (0.71–3.72)	0.255	1.32 (0.58–3.04)	0.510	1.00 (0.42–2.39)	0.995	1.00 (0.40–2.49)	0.998
Group2	6374	7	0.27 (0.13–0.55)	2.49 (1.15–5.39)	0.021	2.08 (0.96–4.52)	0.064	1.67 (0.76–3.69)	0.202	1.67 (0.75–3.74)	0.212
Group3	5257	8	0.36 (0.18–0.70)	3.28 (1.58–6.79)	0.001	2.73 (1.32–5.67)	0.007	2.38 (1.11–5.08)	0.026	2.37 (1.14–4.96)	0.022
Group4	179617	77	0.11 (0.09–0.13)	REF		REF		REF		REF	
**Angina Pectoris**											
Group1	8258	16	0.47 (0.29–0.76)	2.55 (1.52–4.29)	<0.001	2.25 (1.34–3.79)	0.002	1.68 (0.96–2.94)	0.067	1.68 (0.97–2.92)	0.066
Group2	6374	13	0.50 (0.29–0.85)	2.73 (1.55–4.83)	0.001	2.46 (1.39–4.36)	0.002	2.07 (1.15–3.70)	0.015	2.07 (1.14–3.75)	0.017
Group3	5257	12	0.53 (0.31–0.93)	2.90 (1.61–5.24)	<0.001	2.59 (1.43–4.68)	0.002	2.12 (1.14–3.93)	0.017	2.12 (1.12–4.02)	0.022
Group4	179617	130	0.18 (0.15–0.22)	REF		REF		REF		REF	
**Stroke**											
Group1	8258	303	8.83 (7.89–9.88)	0.97 (0.86–1.09)	0.579	1.06 (0.94–1.19)	0.348	1.03 (0.91–1.16)	0.682	1.02 (0.91–1.16)	0.695

Group2	6374	221	8.46 (7.42–9.66)	0.93 (0.81–1.06)	0.275	1.01 (0.88–1.15)	0.903	0.99 (0.86–1.13)	0.859	0.99 (0.86–1.13)	0.852
Group3	5257	210	9.35 (8.17–10.71)	1.03 (0.89–1.18)	0.723	1.11 (0.97–1.27)	0.144	1.07 (0.93–1.24)	0.325	1.07 (0.93–1.24)	0.328
Group4	179617	6520	9.12 (8.90–9.34)	REF		REF		REF		REF	
**Heart Failure**											
Group1	8258	240	6.99 (6.16–7.94)	2.01 (1.76–2.30)	<0.001	1.90 (1.67–2.18)	<0.001	1.41 (1.23–1.63)	<0.001	1.41 (1.22–1.63)	<0.001
Group2	6374	143	5.48 (4.65–6.45)	1.58 (1.33–1.87)	<0.001	1.51 (1.28–1.79)	<0.001	1.27 (1.07–1.51)	0.006	1.27 (1.07–1.51)	0.006
Group3	5257	150	6.68 (5.69–7.84)	1.91 (1.62–2.26)	<0.001	1.82 (1.54–2.15)	<0.001	1.50 (1.26–1.78)	<0.001	1.50 (1.26–1.78)	<0.001
Group4	179617	2471	3.46 (3.32–3.59)	REF		REF		REF		REF	
**Atrial fibrillation**											
Group1	8258	122	3.55 (2.98–4.24)	1.17 (0.98–1.41)	0.083	1.11 (0.93–1.33)	0.259	1.03 (0.85–1.25)	0.767	1.03 (0.85–1.24)	0.775
Group2	6374	93	3.56 (2.91–4.36)	1.18 (0.96–1.45)	0.126	1.13 (0.92–1.39)	0.248	1.10 (0.89–1.36)	0.358	1.10 (0.89–1.36)	0.359
Group3	5257	76	3.38 (2.71–4.24)	1.12 (0.89–1.41)	0.338	1.06 (0.84–1.34)	0.606	1.00 (0.79–1.26)	0.976	1.00 (0.78–1.26)	0.973
Group4	179617	2169	3.03 (2.91–3.16)	REF		REF		REF		REF	

Group1, Low-to-Low; Group2, Low-to-Normal; Group3, Normal-to-Low; Group4, Normal-to-Normal. Model 1 is unadjusted; Model 2 is adjusted for age and sex, as applicable; Model 3 is the fully adjusted multivariable model; Model 4 represents the Fine–Gray competing risk model adjusted for the same covariates as Model 3. Incidence rates are presented per 1,000 person-years with 95% confidence intervals. HDL-C, high-density lipoprotein cholesterol; HR, hazard ratio; sHR, subdistribution hazard ratio; CI, confidence interval.

Table S14. Association between HDL-C trajectory groups and cardiovascular disease risk among participants using lipid-lowering medications, stratified by age: participants aged ≥65 years

				Model1		Model2		Model3		Model4	
	Person	Events	Incidence Rate per 1,000 Person Years (95% CI)	HR (95%CI)	P-value	HR (95%CI)	P-value	HR (95%CI)	P-value	sHR (95%CI)	P-value
**Composite CVD**											
Group1	576	72	42.80 (34.02–53.90)	1.45 (1.14–1.83)	0.002	1.30 (1.02–1.64)	0.032	1.22 (0.96–1.55)	0.109	1.22 (0.95–1.56)	0.111
Group2	459	56	42.02 (32.39–54.56)	1.42 (1.09–1.86)	0.009	1.31 (1.01–1.71)	0.044	1.26 (0.97–1.65)	0.088	1.26 (0.97–1.65)	0.088
Group3	415	53	41.05 (31.42–53.70)	1.39 (1.06–1.82)	0.018	1.25 (0.95–1.65)	0.104	1.18 (0.89–1.55)	0.256	1.17 (0.89–1.55)	0.262
Group4	23912	2097	29.50 (28.26–30.79)	REF		REF		REF		REF	
**Myocardial Infarction**											
Group1	576	0	0.00 (0.02–2.19)	0.00 (0.00–.)	0.995	0.00 (0.00–.)	0.995	0.00 (0.00–.)	0.997	0.00 (0.00–0.00)	<0.001
Group2	459	0	0.00 (0.02–2.77)	0.00 (0.00–.)	0.995	0.00 (0.00–.)	0.996	0.00 (0.00–.)	0.997	0.00 (0.00–0.00)	<0.001
Group3	415	0	0.00 (0.02–2.86)	0.00 (0.00–.)	0.995	0.00 (0.00–.)	0.996	0.00 (0.00–.)	0.997	0.00 (0.00–0.00)	<0.001
Group4	23912	12	0.17 (0.10–0.29)	REF		REF		REF		REF	
**Angina Pectoris**											
Group1	576	2	1.19 (0.37–4.29)	4.66 (1.08–20.1)	0.039	3.02 (0.70–13.1)	0.139	1.75 (0.38–8.07)	0.470	1.76 (0.39–7.98)	0.464
Group2	459	0	0.00 (0.02–2.77)	0.00 (0.00–.)	0.991	0.00 (0.00–.)	0.993	0.00 (0.00–.)	0.999	0.00 (0.00–0.00)	<0.001
Group3	415	0	0.00 (0.02–2.86)	0.00 (0.00–0.00)	0.991	0.00 (0.00–0.00)	0.993	0.00 (0.00–0.00)	0.997	0.00 (0.00–0.00)	<0.001
Group4	23912	18	0.25 (0.16–0.40)	REF		REF		REF		REF	
**Stroke**											
Group1	576	26	15.46 (10.58–22.65)	0.97 (0.66–1.44)	0.894	0.99 (0.67–1.46)	0.945	0.98 (0.66–1.47)	0.936	0.98 (0.66–1.47)	0.930

Group2	459	27	20.26 (13.96–29.47)	1.28 (0.87–1.87)	0.207	1.30 (0.89–1.91)	0.177	1.31 (0.89–1.93)	0.171	1.31 (0.89–1.93)	0.167
Group3	415	16	12.39 (7.67–20.13)	0.78 (0.48–1.28)	0.326	0.79 (0.48–1.29)	0.344	0.77 (0.47–1.28)	0.319	0.77 (0.47–1.27)	0.312
Group4	23912	1124	15.81 (14.91–16.76)	REF		REF		REF		REF	
**Heart Failure**											
Group1	576	21	12.48 (8.20–19.08)	2.01 (1.30–3.12)	0.002	1.63 (1.05–2.53)	0.030	1.34 (0.85–2.10)	0.211	1.34 (0.85–2.11)	0.212
Group2	459	14	10.50 (6.30–17.62)	1.70 (1.00–2.89)	0.051	1.44 (0.85–2.46)	0.178	1.25 (0.73–2.14)	0.419	1.25 (0.73–2.14)	0.413
Group3	415	20	15.49 (10.07–23.93)	2.49 (1.59–3.89)	<0.001	2.05 (1.31–3.22)	0.002	1.70 (1.07–2.71)	0.024	1.70 (1.07–2.69)	0.025
Group4	23912	439	6.17 (5.62–6.78)	REF		REF		REF		REF	
**Atrial fibrillation**											
Group1	576	23	13.67 (9.14–20.52)	1.93 (1.27–2.93)	0.002	1.51 (0.99–2.30)	0.054	1.46 (0.94–2.25)	0.089	1.46 (0.95–2.24)	0.086
Group2	459	15	11.25 (6.86–18.56)	1.59 (0.95–2.67)	0.075	1.32 (0.79–2.21)	0.286	1.29 (0.77–2.17)	0.332	1.30 (0.77–2.19)	0.333
Group3	415	17	13.17 (8.26–21.08)	1.87 (1.15–3.03)	0.011	1.51 (0.93–2.44)	0.098	1.44 (0.88–2.36)	0.152	1.43 (0.87–2.36)	0.155
Group4	23912	504	7.09 (6.50–7.74)	REF		REF		REF		REF	

Group1, Low-to-Low; Group2, Low-to-Normal; Group3, Normal-to-Low; Group4, Normal-to-Normal. Model 1 is unadjusted; Model 2 is adjusted for age and sex, as applicable; Model 3 is the fully adjusted multivariable model; Model 4 represents the Fine–Gray competing risk model adjusted for the same covariates as Model 3. Incidence rates are presented per 1,000 person-years with 95% confidence intervals. HDL-C, high-density lipoprotein cholesterol; HR, hazard ratio; sHR, subdistribution hazard ratio; CI, confidence interval.

Table S15. Sensitivity analysis without excluding participants with follow-up shorter than 1 year

	Person	Events	Incidence Rate per 1,000 Person Years (95% CI)	Model1		Model2		Model3	
				HR (95%CI)	P-value	HR (95%CI)	P-value	HR (95%CI)	P-value
**Composite CVD**									
Group1	92617	4507	12.04 (11.69–12.39)	1.40 (1.36–1.45)	<0.001	1.33 (1.29–1.37)	<0.001	1.16 (1.12–1.20)	<0.001
Group2	70235	3034	10.69 (10.32–11.08)	1.25 (1.20–1.29)	<0.001	1.21 (1.16–1.25)	<0.001	1.10 (1.06–1.14)	<0.001
Group3	69161	3089	11.09 (10.71–11.49)	1.29 (1.25–1.34)	<0.001	1.28 (1.23–1.32)	<0.001	1.14 (1.10–1.19)	<0.001
Group4	3512535	115896	8.57 (8.52–8.62)	REF		REF		REF	
**Myocardial Infarction**									
Group1	92617	70	0.19 (0.15–0.24)	5.62 (4.37–7.24)	<0.001	4.02 (3.12–5.18)	<0.001	2.13 (1.61–2.80)	<0.001
Group2	70235	31	0.11 (0.08–0.16)	3.28 (2.28–4.72)	<0.001	2.48 (1.72–3.57)	<0.001	1.42 (0.97–2.06)	0.069
Group3	69161	32	0.11 (0.08–0.16)	3.45 (2.41–4.93)	<0.001	2.67 (1.87–3.83)	<0.001	1.65 (1.13–2.39)	0.009
Group4	3512535	449	0.03 (0.03–0.04)	REF		REF		REF	
**Angina Pectoris**									
Group1	92617	83	0.22 (0.18–0.27)	4.22 (3.36–5.30)	<0.001	3.05 (2.42–3.83)	<0.001	1.73 (1.36–2.21)	<0.001
Group2	70235	50	0.18 (0.13–0.23)	3.36 (2.52–4.47)	<0.001	2.57 (1.93–3.42)	<0.001	1.57 (1.16–2.11)	0.003
Group3	69161	51	0.18 (0.14–0.24)	3.48 (2.62–4.63)	<0.001	2.74 (2.06–3.64)	<0.001	1.75 (1.30–2.35)	<0.001
Group4	3512535	707	0.05 (0.05–0.06)	REF		REF		REF	
**Stroke**									
Group1	92617	2313	6.18 (5.93–6.43)	1.10 (1.06–1.15)	<0.001	1.14 (1.09–1.18)	<0.001	1.04 (0.99–1.08)	0.109
Group2	70235	1722	6.07 (5.79–6.36)	1.09 (1.04–1.14)	0.001	1.13 (1.08–1.19)	<0.001	1.06 (1.01–1.11)	0.019
Group3	69161	1708	6.13 (5.85–6.43)	1.10 (1.05–1.15)	<0.001	1.16 (1.11–1.22)	<0.001	1.07 (1.02–1.13)	0.004
Group4	3512535	75479	5.58 (5.54–5.62)	REF		REF		REF	
**Heart Failure**									
Group1	92617	1257	3.36 (3.18–3.55)	2.27 (2.15–2.41)	<0.001	1.96 (1.85–2.07)	<0.001	1.44 (1.35–1.53)	<0.001
Group2	70235	727	2.56 (2.38–2.76)	1.73 (1.61–1.86)	<0.001	1.54 (1.43–1.66)	<0.001	1.24 (1.15–1.34)	<0.001
Group3	69161	775	2.78 (2.59–2.99)	1.88 (1.75–2.02)	<0.001	1.71 (1.59–1.84)	<0.001	1.36 (1.26–1.46)	<0.001

Group4	3512535	19903	1.47 (1.45–1.49)	REF		REF		REF	
**Atrial fibrillation**									
Group1	92617	784	2.09 (1.95–2.25)	1.46 (1.36–1.57)	<0.001	1.18 (1.10–1.27)	<0.001	1.10 (1.02–1.19)	0.013
Group2	70235	504	1.78 (1.63–1.94)	1.24 (1.14–1.36)	<0.001	1.05 (0.96–1.14)	0.308	1.01 (0.93–1.11)	0.801
Group3	69161	523	1.88 (1.72–2.05)	1.31 (1.20–1.43)	<0.001	1.14 (1.04–1.24)	0.004	1.07 (0.98–1.17)	0.157
Group4	3512535	19358	1.43 (1.41–1.45)	REF		REF		REF	

Group1, Low-to-Low; Group2, Low-to-Normal; Group3, Normal-to-Low; Group4, Normal-to-Normal. Model 1 is unadjusted; Model 2 is adjusted for age and sex, as applicable; Model 3 is the fully adjusted multivariable model. Incidence rates are presented per 1,000 person-years with 95% confidence intervals. HDL-C, high-density lipoprotein cholesterol; HR, hazard ratio; CI, confidence interval.

Table S16. Sensitivity analysis with additional adjustment for eGFR

				Model1		Model2		Model3	
	Person	Events	Incidence Rate per 1,000 Person Years (95% CI)	HR (95%CI)	P-value	HR (95%CI)	P-value	HR (95%CI)	P-value
**Composite CVD**									
Group1	43823	2073	11.34 (10.86–11.84)	1.40 (1.34–1.46)	<0.001	1.31 (1.25–1.37)	<0.001	1.14 (1.09–1.19)	<0.001
Group2	32822	1429	10.44 (9.91–10.99)	1.28 (1.22–1.35)	<0.001	1.23 (1.17–1.30)	<0.001	1.12 (1.06–1.18)	<0.001
Group3	31878	1425	10.68 (10.14–11.25)	1.31 (1.25–1.39)	<0.001	1.29 (1.23–1.36)	<0.001	1.17 (1.11–1.23)	<0.001
Group4	1581437	52306	8.12 (8.05–8.19)	REF		REF		REF	
**Myocardial Infarction**									
Group1	43823	33	0.18 (0.13–0.25)	6.96 (4.79–10.1)	<0.001	4.80 (3.30–6.98)	<0.001	2.85 (1.89–4.30)	<0.001
Group2	32822	13	0.09 (0.06–0.16)	3.66 (2.08–6.43)	<0.001	2.67 (1.52–4.70)	0.001	1.61 (0.90–2.88)	0.110
Group3	31878	15	0.11 (0.07–0.19)	4.33 (2.55–7.34)	<0.001	3.25 (1.92–5.52)	<0.001	2.26 (1.30–3.91)	0.004
Group4	1581437	167	0.03 (0.02–0.03)	REF		REF		REF	
**Angina Pectoris**									
Group1	43823	36	0.20 (0.14–0.27)	4.27 (3.02–6.03)	<0.001	2.99 (2.11–4.23)	<0.001	1.59 (1.09–2.31)	0.015
Group2	32822	26	0.19 (0.13–0.28)	4.13 (2.77–6.17)	<0.001	3.08 (2.06–4.61)	<0.001	1.84 (1.21–2.78)	0.004
Group3	31878	24	0.18 (0.12–0.27)	3.90 (2.57–5.91)	<0.001	3.02 (1.99–4.58)	<0.001	1.85 (1.19–2.86)	0.006
Group4	1581437	296	0.05 (0.04–0.05)	REF		REF		REF	
**Stroke**									
Group1	43823	1059	5.79 (5.45–6.15)	1.09 (1.03–1.16)	0.006	1.11 (1.04–1.18)	0.001	1.01 (0.95–1.08)	0.748
Group2	32822	826	6.03 (5.64–6.46)	1.14 (1.06–1.22)	<0.001	1.17 (1.09–1.25)	<0.001	1.09 (1.02–1.17)	0.012
Group3	31878	804	6.02 (5.62–6.46)	1.13 (1.06–1.22)	<0.001	1.19 (1.11–1.28)	<0.001	1.11 (1.03–1.19)	0.007
Group4	1581437	34197	5.31 (5.25–5.36)	REF		REF		REF	
**Heart Failure**									
Group1	43823	586	3.21 (2.96–3.48)	2.32 (2.14–2.52)	<0.001	1.99 (1.83–2.17)	<0.001	1.43 (1.31–1.57)	<0.001
Group2	32822	346	2.53 (2.27–2.81)	1.83 (1.64–2.03)	<0.001	1.63 (1.46–1.81)	<0.001	1.29 (1.16–1.44)	<0.001
Group3	31878	319	2.39 (2.14–2.67)	1.73 (1.55–1.93)	<0.001	1.58 (1.41–1.77)	<0.001	1.25 (1.11–1.40)	<0.001

Group4	1581437	8883	1.38 (1.35–1.41)	REF		REF		REF	
**Atrial fibrillation**									
Group1	43823	359	1.96 (1.77–2.18)	1.44 (1.30–1.61)	<0.001	1.15 (1.04–1.28)	0.009	1.08 (0.97–1.21)	0.154
Group2	32822	218	1.59 (1.39–1.82)	1.17 (1.02–1.34)	0.022	0.98 (0.86–1.12)	0.761	0.95 (0.83–1.09)	0.483
Group3	31878	263	1.97 (1.75–2.22)	1.45 (1.28–1.64)	<0.001	1.26 (1.11–1.42)	<0.001	1.20 (1.06–1.36)	0.005
Group4	1581437	8763	1.36 (1.33–1.39)	REF		REF		REF	

Group1, Low-to-Low; Group2, Low-to-Normal; Group3, Normal-to-Low; Group4, Normal-to-Normal. Model 1 is unadjusted; Model 2 is adjusted for age and sex, as applicable; Model 3 is the fully adjusted multivariable model. Incidence rates are presented per 1,000 person-years with 95% confidence intervals. HDL-C, high-density lipoprotein cholesterol; HR, hazard ratio; CI, confidence interval.

Table S17. Sensitivity analysis using a complete-case approach excluding participants with missing categorical covariates

				Model1		Model2		Model3	
	Person	Events	Incidence Rate per 1,000 Person Years (95% CI)	HR (95%CI)	P-value	HR (95%CI)	P-value	HR (95%CI)	P-value
**Composite CVD**									
Group1	75606	3933	12.16 (11.79–12.55)	1.40 (1.35–1.44)	<0.001	1.32 (1.27–1.36)	<0.001	1.14 (1.11–1.18)	<0.001
Group2	57317	2684	10.92 (10.52–11.35)	1.25 (1.20–1.30)	<0.001	1.21 (1.16–1.26)	<0.001	1.10 (1.06–1.14)	<0.001
Group3	55473	2684	11.28 (10.86–11.72)	1.29 (1.24–1.34)	<0.001	1.27 (1.22–1.32)	<0.001	1.13 (1.09–1.18)	<0.001
Group4	2844320	102128	8.71 (8.65–8.76)	REF		REF		REF	
**Myocardial Infarction**									
Group1	75606	68	0.21 (0.17–0.27)	6.48 (5.00–8.38)	<0.001	4.62 (3.56–5.98)	<0.001	2.34 (1.76–3.11)	<0.001
Group2	57317	25	0.10 (0.07–0.15)	3.13 (2.09–4.68)	<0.001	2.36 (1.57–3.54)	<0.001	1.33 (0.88–2.02)	0.177
Group3	55473	26	0.11 (0.07–0.16)	3.36 (2.26–4.99)	<0.001	2.58 (1.73–3.84)	<0.001	1.53 (1.02–2.32)	0.041
Group4	2844320	381	0.03 (0.03–0.04)	REF		REF		REF	
**Angina Pectoris**									
Group1	75606	68	0.21 (0.17–0.27)	3.84 (2.99–4.93)	<0.001	2.76 (2.15–3.55)	<0.001	1.54 (1.18–2.02)	0.002
Group2	57317	47	0.19 (0.14–0.25)	3.49 (2.59–4.69)	<0.001	2.66 (1.98–3.58)	<0.001	1.61 (1.18–2.19)	0.002
Group3	55473	45	0.19 (0.14–0.25)	3.44 (2.54–4.66)	<0.001	2.67 (1.97–3.61)	<0.001	1.68 (1.23–2.31)	0.001
Group4	2844320	640	0.05 (0.05–0.06)	REF		REF		REF	
**Stroke**									
Group1	75606	1997	6.17 (5.91–6.45)	1.09 (1.04–1.14)	<0.001	1.12 (1.07–1.17)	<0.001	1.01 (0.97–1.06)	0.543
Group2	57317	1503	6.12 (5.82–6.43)	1.08 (1.02–1.14)	0.004	1.12 (1.06–1.18)	<0.001	1.05 (1.00–1.10)	0.070
Group3	55473	1482	6.23 (5.92–6.56)	1.10 (1.04–1.16)	<0.001	1.16 (1.10–1.22)	<0.001	1.07 (1.01–1.12)	0.019
Group4	2844320	66381	5.66 (5.62–5.70)	REF		REF		REF	
**Heart Failure**									
Group1	75606	1118	3.46 (3.26–3.67)	2.30 (2.16–2.44)	<0.001	1.97 (1.85–2.09)	<0.001	1.43 (1.34–1.52)	<0.001
Group2	57317	658	2.68 (2.48–2.89)	1.77 (1.64–1.92)	<0.001	1.57 (1.46–1.70)	<0.001	1.26 (1.16–1.36)	<0.001
Group3	55473	676	2.84 (2.64–3.06)	1.88 (1.74–2.03)	<0.001	1.70 (1.57–1.83)	<0.001	1.33 (1.23–1.44)	<0.001

Group4	2844320	17585	1.50 (1.48–1.52)	REF		REF		REF	
**Atrial fibrillation**									
Group1	75606	682	2.11 (1.96–2.27)	1.45 (1.34–1.56)	<0.001	1.16 (1.08–1.25)	<0.001	1.10 (1.02–1.19)	0.020
Group2	57317	451	1.84 (1.67–2.01)	1.25 (1.14–1.38)	<0.001	1.06 (0.96–1.16)	0.234	1.03 (0.94–1.13)	0.515
Group3	55473	455	1.91 (1.75–2.10)	1.31 (1.19–1.44)	<0.001	1.12 (1.02–1.23)	0.014	1.07 (0.97–1.18)	0.167
Group4	2844320	17141	1.46 (1.44–1.48)	REF		REF		REF	

Group1, Low-to-Low; Group2, Low-to-Normal; Group3, Normal-to-Low; Group4, Normal-to-Normal. Model 1 is unadjusted; Model 2 is adjusted for age and sex, as applicable; Model 3 is the fully adjusted multivariable model. Incidence rates are presented per 1,000 person-years with 95% confidence intervals. HDL-C, high-density lipoprotein cholesterol; HR, hazard ratio; CI, confidence interval.

Figure S1. Log-minus-log survival plot for composite cardiovascular disease according to HDL-C trajectory groups.

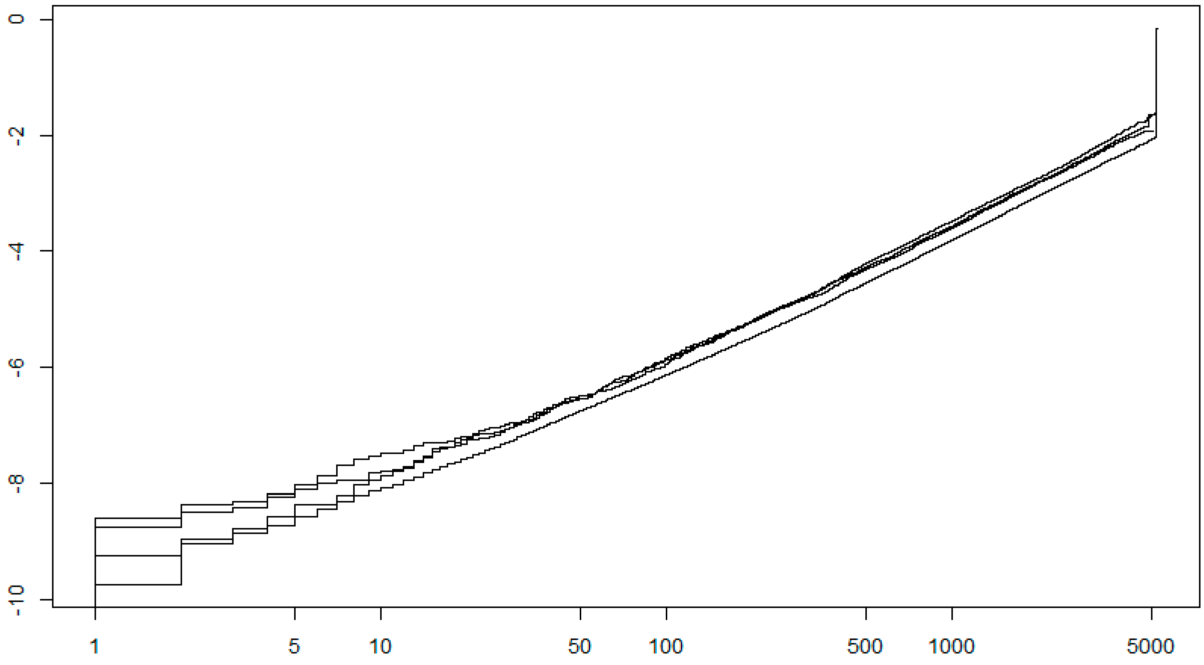

The generally parallel pattern of the curves suggests no substantial violation of the proportional hazards assumption for the primary outcome.

Figure S2. Kaplan–Meier curves for cumulative incidence of cardiovascular outcomes among participants with lipid-lowering medication use

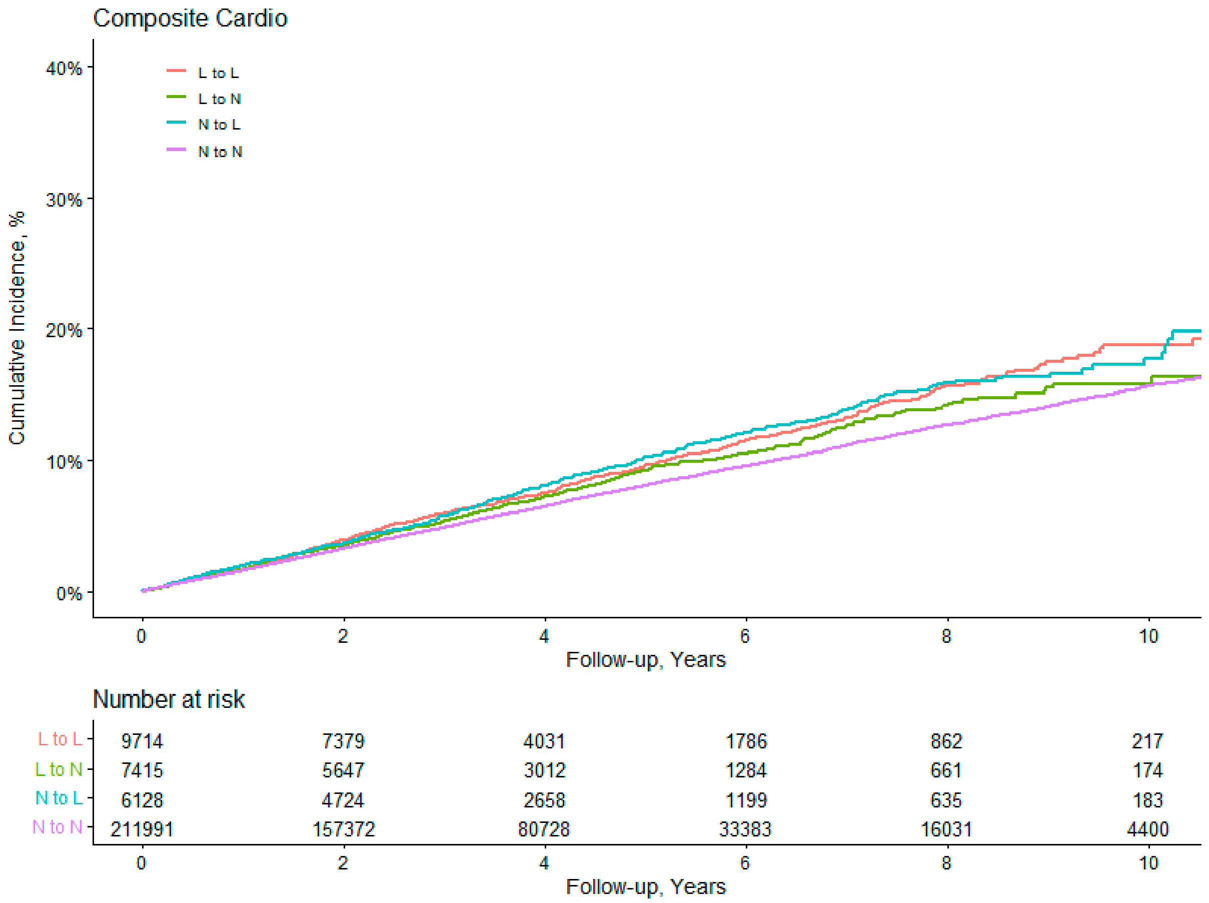

Figure S3. Kaplan–Meier curves for cumulative incidence of cardiovascular outcomes among participants with lipid-lowering medication use, stratified by sex: male participants

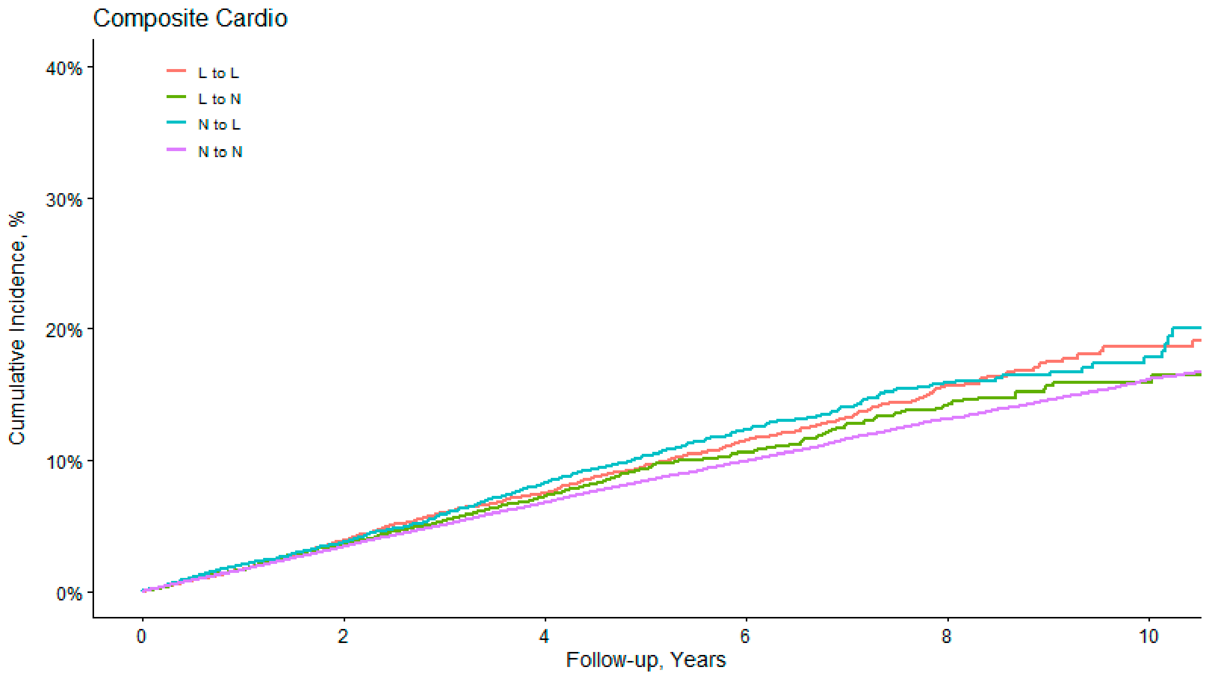

Number at risk

	0	2	4	6	8	10
L to L	9124	6963	3815	1704	824	210
L to N	6716	5141	2784	1207	634	164
N to L	5555	4311	2465	1128	599	175
N to N	141620	107494	58012	25067	12602	3645

Follow-up, Years

Figure S4. Kaplan–Meier curves for cumulative incidence of cardiovascular outcomes among participants with lipid-lowering medication use, stratified by sex: female participants

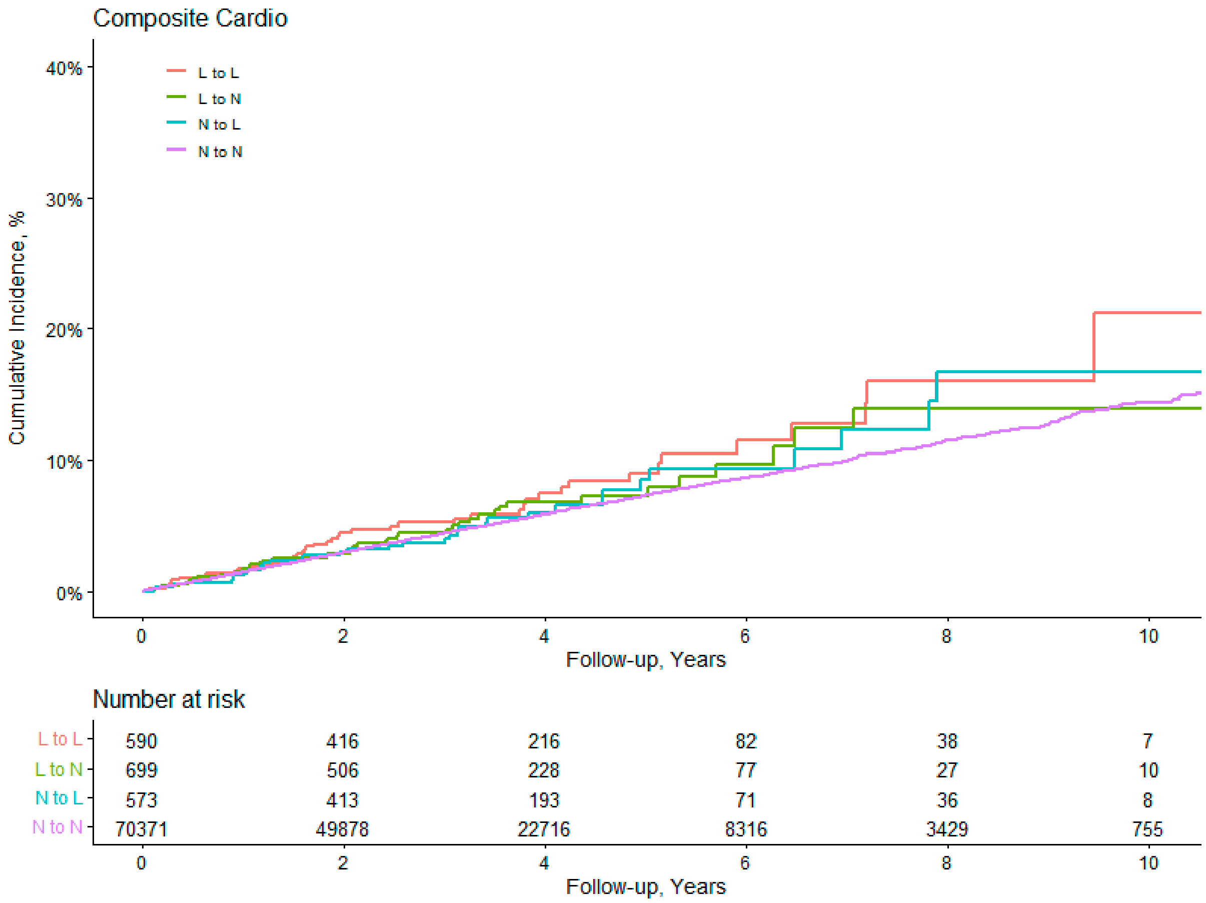

Figure 5. Kaplan–Meier curves for cumulative incidence of cardiovascular outcomes among participants with lipid-lowering medication use, stratified by age: participants aged <40 years

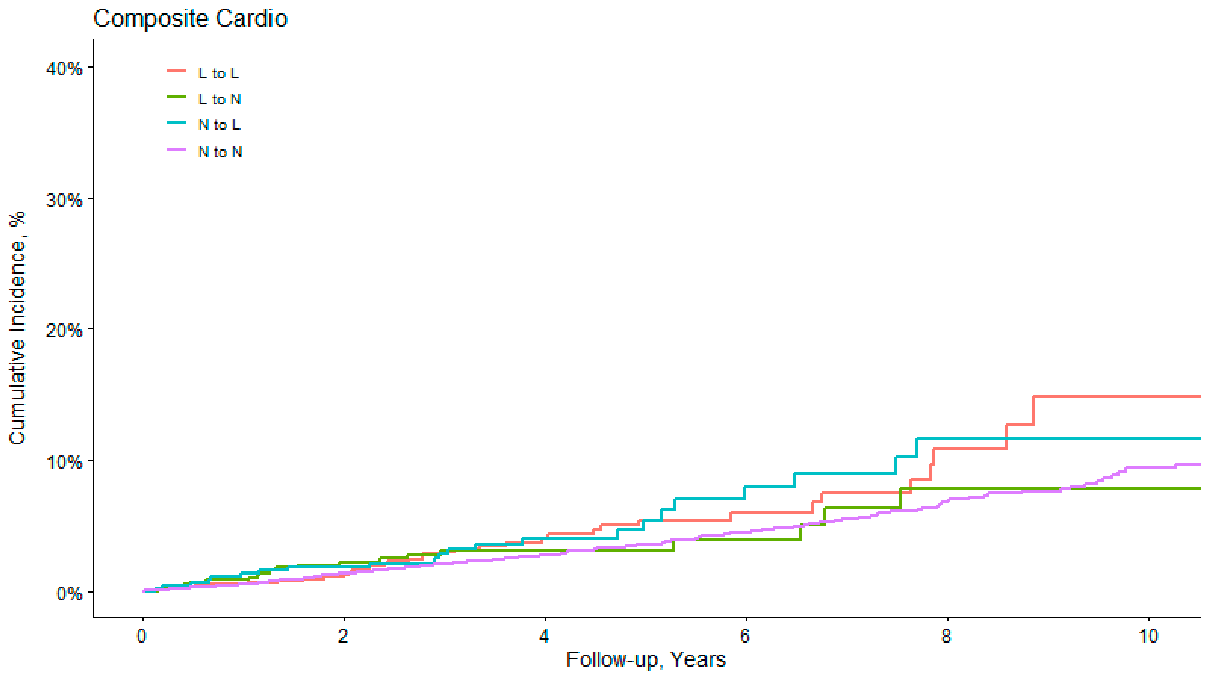

	0	2	4	6	8	10
L to L	880	625	324	152	69	21
L to N	582	399	208	95	54	19
N to L	456	333	184	100	58	16
N to N	8462	5896	2979	1444	806	339

Follow-up, Years

Figure 6. Kaplan–Meier curves for cumulative incidence of cardiovascular outcomes among participants with lipid-lowering medication use, stratified by age: participants aged 40-64 years

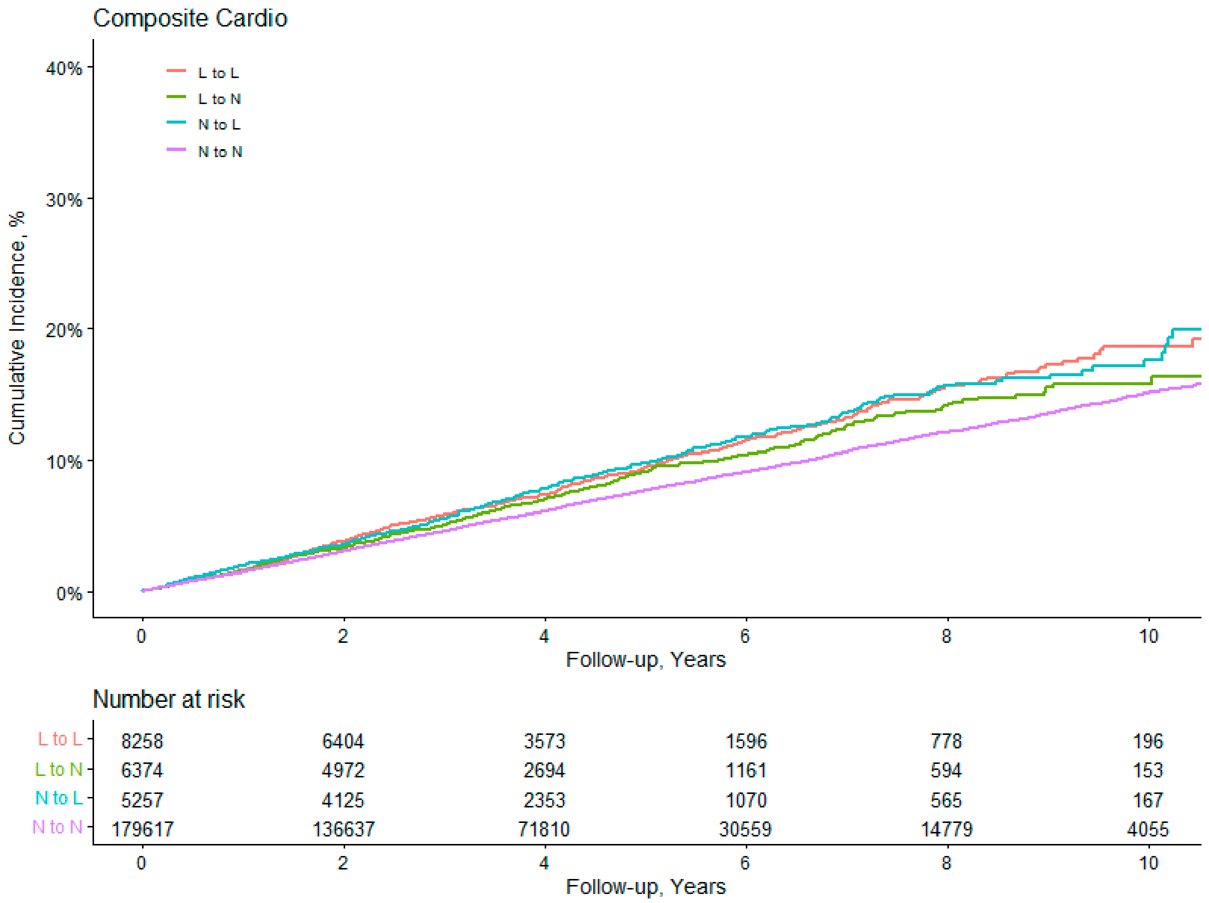

Figure 7. Kaplan–Meier curves for cumulative incidence of cardiovascular outcomes among participants with lipid-lowering medication use, stratified by age: participants aged ≥65 years

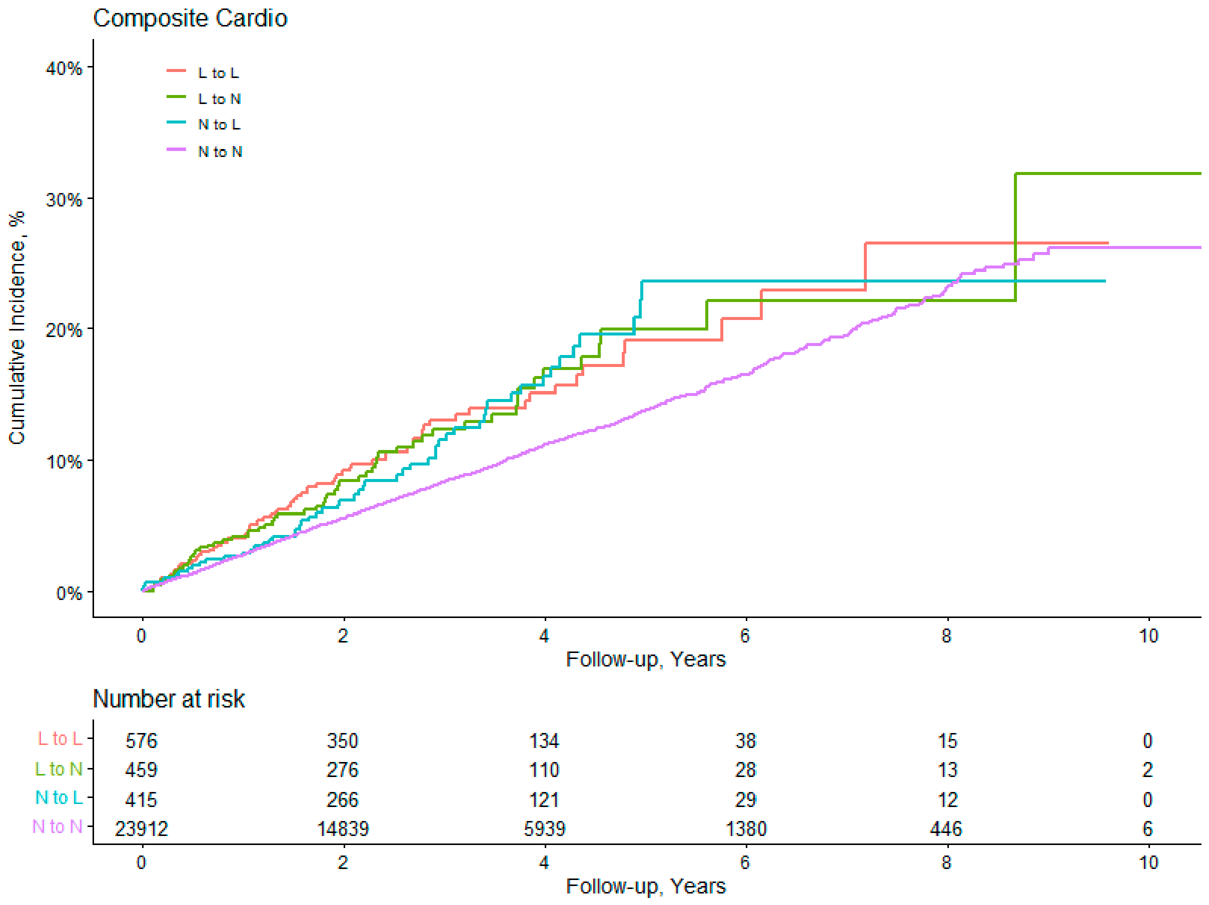

Supplement: Supplementary file 1 [file healthcare-14-01959-s001.zip › Supplementary_materials_JP.pdf]
